# Patient Safety in the Eyes of Aspiring Healthcare Professionals: A Systematic Review of Their Attitudes

**DOI:** 10.3390/ijerph18147524

**Published:** 2021-07-15

**Authors:** Ilaria Tocco Tussardi, Roberto Benoni, Francesca Moretti, Stefano Tardivo, Albino Poli, Albert W. Wu, Michela Rimondini, Isolde Martina Busch

**Affiliations:** 1 Department of Diagnostics and Public Health, University of Verona, 37134 Verona, Italy; ilaria.toccotussardi@studenti.univr.it (I.T.T.); roberto.benoni90@gmail.com (R.B.); stefano.tardivo@univr.it (S.T.); albino.poli@univr.it (A.P.); 2 Department of Neuroscience, Biomedicine and Movement Sciences, University of Verona, 37134 Verona, Italy; francesca.moretti76@gmail.com (F.M.); isoldemartina.busch@univr.it (I.M.B.); 3 Department of Health Policy and Management, Johns Hopkins Bloomberg School of Public Health, Baltimore, MD 21205, USA; awu@jhu.edu

**Keywords:** patient safety culture, safety culture, attitudes, young healthcare professionals, junior doctors

## Abstract

A culture of safety is important for the delivery of safe, high-quality care, as well as for healthcare providers’ wellbeing. This systematic review aimed to describe and synthesize the literature on patient safety attitudes of the next generation of healthcare workers (health professional students, new graduates, newly registered health professionals, resident trainees) and assess potential differences in this population related to years of study, specialties, and gender. We screened four electronic databases up to 20 February 2020 and additional sources, including weekly e-mailed search alerts up to 18 October 2020. Two independent reviewers conducted the search, study selection, quality rating, data extraction, and formal narrative synthesis, involving a third reviewer in case of dissent. We retrieved 6606 records, assessed 188 full-texts, and included 31 studies. Across articles, healthcare students and young professionals showed overwhelmingly positive patient safety attitudes in some areas (e.g., teamwork climate, error inevitability) but more negative perceptions in other domains (e.g., safety climate, disclosure responsibility). Women tend to report more positive attitudes. To improve safety culture in medical settings, health professions educators and institutions should ensure education and training on patient safety.

## 1. Introduction

Studies from the past two decades indicate that medical error is a leading cause of death in both the United States and the European Union [1,2]. Medical error, defined by the US National Academy of Medicine (formerly the Institute of Medicine) as “the failure of a planned action to be completed as intended or the use of a wrong plan to achieve an aim”, is widely understood to be caused by system rather than individual factors [3].

Medical errors are also harmful for healthcare workers who frequently experience negative psychological effects such as guilt, anxiety, anger, depression, and feelings of inadequacy [4]. Given the broad adverse impact of medical errors on patients, their caregivers, and healthcare workers [5,6], their prevention must be a top priority for national and local health systems. Creating a safety culture in healthcare organizations has been recognized as a key strategy for ensuring patient safety, reducing medical errors, and improving the quality of care [7].

Safety culture is “the product of individual and group values, attitudes, perceptions, competencies, and patterns of behavior that determine the commitment to, and the style and proficiency of, an organization’s health and safety management” (p. 156) [8]. Safety climate is a measurable component of safety culture, derived from the attitudes and perceptions of individuals that are part of a group (e.g., unit, service, department, or organization) at a given time [9]. Questionnaires and surveys are generally used to measure safety climate at the individual and group level. Since health care is usually provided by teams of professionals working within a larger unit or organization, the attitudes and behaviors of the entire group are of particular interest [10].

Health professional students are an important group. Developing and enhancing the attitudes of the next generation of healthcare workers towards safety culture, and enhancing the related skills, knowledge, and safety behaviors will help to create safer healthcare settings in the future [11,12,13]. Attitudes are the basis for appropriate safety culture and climate. Knowledge, skills, and behaviors are built on them and allow the understanding and adherence to safety guidelines [14]. The World Health Organization (WHO) has endorsed introducing the concept of safety culture during the training of future healthcare workers [13,15,16]. In 2010, the WHO developed a patient safety curriculum for medical schools [15,16].

While these studies [11,12,13,14] emphasize the importance of instilling attitudes related to safety culture in aspiring healthcare professionals, no study has been found which systematically reviewed the existing literature on this topic. Thus, this systematic review aimed to describe and synthesize the literature on patient safety attitudes of health professional students, new graduates, newly registered health professionals, and resident trainees. This study also seeks to assess differences in this population related to years of study, specialty, and gender.

## 2. Materials and Methods

The study protocol is listed in the International Prospective Register of Systematic Reviews (PROSPERO), Registration Number CRD42020211187. After the publication of the registered protocol on the Prospero website on 29 October 2020, we slightly updated the record once, on 19 January 2021 (see *Revision Notes* section of PROSPERO study protocol).

### 2.1. Search and Selection of Studies

The search and selection process followed the guidelines of the Reporting Items for Systematic Reviews and Meta-Analyses (PRISMA) [17].

We conducted a systematic search of four electronic databases (PubMed, Web of Science, Scopus, PsycInfo) up to 20 February 2020 without restriction to language and publication date, applying the following search strategy: (patient safety OR patient safety attitude OR safety climate OR patient safety competence OR patient safety culture OR patient safety values OR patient safety behaviour) AND (students OR junior doctors OR newly graduates OR newly registered OR postgraduate trainees OR resident trainees) (see Supplementary File 1). We did not use a controlled vocabulary but only free-text terms. To detect additional studies, we screened the reference lists of previously published reviews and two grey literature databases (OpenGrey database, Grey Literature project) and set up weekly emailed search alerts for PubMed between 18 February 2020 and 18 October 2020 (see Supplementary File 2).

Original research articles were included if (1) they provided quantitative data on patient safety attitudes among health professional students, new graduates, newly registered health professionals (from all healthcare study courses), and junior doctors (specialty and general practice trainees); (2) the instrument/methodology used to collect the data (e.g., scale/questionnaire/survey) was clarified and described in detail in the study (3) they were published in English, Italian, or German (languages spoken by the authors).

The following types of articles were excluded: quasi-experimental or experimental studies assessing quality improvement/patient safety interventions and applying a pre-post design to measure young healthcare professionals’ attitudes (modified in the latest version of the Prospero protocol updated on 19 January 2021), validation studies, opinion papers/commentaries, editorials, letters, qualitative studies, literature reviews (e.g., narrative reviews, scoping reviews, systematic reviews), book chapters, and theses.

Two independent reviewers (I.T.T. and R.B.) screened titles and abstracts of the records, using the Web application Rayyan [18] and independently evaluated the full texts of records considered as eligible by at least one of them. In case of disagreement, a third reviewer (F.M.) was involved.

### 2.2. Risk of Bias Assessment of the Included Studies

The quality of the included studies was assessed by two appraisers (I.T.T. and R.B.) using the Mixed Methods Appraisal Tool (MMAT) Version 18 [19] (modified in the latest version of the Prospero protocol updated on 19 January 2021). We chose the MMAT as it represents an appropriate tool to assess the quality of the included studies (i.e., quantitative descriptive studies). The risk of bias assessment was based on five quality criteria, namely relevance of the sampling strategy, sample representativeness of the target population, appropriateness of applied measurements, risk of nonresponse bias, and appropriateness of statistical analysis. Any potential dissent was addressed and, if necessary, a third appraiser (I.M.B.) involved.

### 2.3. Data Extraction and Synthesis

After extracting study characteristics (e.g., country, population, sample size, type, and version of questionnaire) and main findings of the included studies (i.e., quantitative data on safety culture attitudes, such as mean scores, subscores, percentage of agreement) using Microsoft Excel, we performed a formal narrative synthesis composed of the following elements:

- Tabular description of the included studies, presented as supplementary material;
- Synthesis of students and young healthcare professionals’ attitudes assessed by (1) the Safety Attitudes Questionnaires (SAQ), (2) the Attitudes to Patient Safety Questionnaire (APSQ), (3) Hospital Survey on Patient Safety Culture (HSOPSC) by the Agency for Healthcare Research and Quality (AHRQ), (4) other questionnaires, structured around areas with regard to awareness/perception of patient safety culture, presented as figure, narrative text, and supplementary material;
- Synthesis of reported differences (1) across years of study, (2) across specialties, (3) between genders, presented as table and narrative text;
- When synthesizing the data, we followed some specific guidelines:
- For articles using the SAQ, APSQ, or HSOPSC, if only the mean item scores were given, we calculated the mean domain scores with Excel, taking into account reverse scored items. The same procedure was applied for percentages of positive answers.
- For articles using the SAQ, we followed the recommendations given in the literature [20,21,22] and adopted a cut-off point of ≥75 for SAQ mean scores. Namely, we considered SAQ mean scores of ≥75 as “high”. Consequently, we considered SAQ mean scores of ≥60 and <75 as “acceptable”.
- For the articles applying the APSQ, we did not compare the reported mean scores of the domains across but only within studies since different types of Likert scales (e.g., 5 or 7 points) were used across studies. Based on the indications in the literature [23,24,25,26], responses to individual items rated with 7-point Likert scales were considered as a positive/desired attitude if the response was “strongly agree”, “agree” or “somewhat agree” in positively worded questions and “strongly disagree”, “disagree” and “somewhat disagree” in negatively worded questions and mean scores of 5-point Likert scales were considered as a positive/desired response if the response was “strongly agree” or “agree” in positively worded questions and “strongly disagree” or “disagree” in negatively worded questions. Consequently, mean scores of domains assessed by a 7-point Likert scale reflect a more positive/desired attitude if >4, while mean scores measured by a 5-point Likert scale point to a more positive/desired attitude if >3.
- For the articles using the HSOPSC, for two of the three studies [27,28] the percentages of respondents answering positively were estimated on the basis of the tables presented, which did not show the exact value of the percentages.

## 3. Results

The search of the electronic databases and additional sources initially produced 6606 records (without duplicates). After screening title and/or abstract, 188 full-text articles were assessed for eligibility. Of these, 157 studies were excluded for various reasons, such as mismatch with the inclusion criteria, wrong study design (e.g., validation study, quasi-experimental design), mixed population, wrong focus of the study, or full text not available (see Supplementary File 3). Finally, 31 studies meeting the inclusion criteria were included [13,20,22,23,24,25,26,27,28,29,30,31,32,33,34,35,36,37,38,39,40,41,42,43,44,45,46,47,48,49,50] (see Supplementary File 4).

### 3.1. Risk of Bias Assessment of the Included Studies

Seventeen studies fulfilled at least four of the five quality appraisal criteria [22,24,25,26,27,28,30,31,33,35,37,38,39,40,43,45,48], and five studies met all criteria [28,35,37,39,45].

In all included studies, the sampling strategy was relevant to address the research question. In more than two thirds of the studies, the study sample was clearly described and representative of the target population [13,20,23,24,25,26,28,30,31,32,33,34,35,37,38,39,40,42,43,45,47,48,49,50], the risk of non-response bias low [22,23,24,25,26,27,28,30,31,33,35,36,39,40,41,42,43,44,45,46,48,49], and the statistical analysis appropriate to answer the research question [13,20,22,24,25,27,28,29,30,31,32,33,34,35,36,37,38,39,43,44,45,46,47,48]. While most studies used instruments, which were validated in their original language

[13,20,22,23,24,25,26,27,28,30,35,37,38,39,40,42,44,45,48], it remained unclear for several studies if the respective questionnaires had also been validated in the language in which they had been administered [20,23,24,25,30,42,44,48].

Supplementary File 5 offers a comprehensive overview of appraisers’ judgements.

### 3.2. Characteristics of the Included Studies

The 31 included studies, all of which were written in English, were published between 2005 and 2020. The years 2013 and 2018 showed the highest number of publications [24,31,33,34,36,39,42,49,50]. With regard to the countries of origin, very different geographical and cultural areas were represented, such as the US (9 studies) [26,27,28,31,36,40,45,47,49], the Middle East and Indian sub-continent (four published in Saudi Arabia [13,22,29,30], four in Pakistan [23,24,25,46], one in Iran [43]), East Asia (two in China [38,42], one in Hong Kong [41], two in South Korea [39,44], one in Malaysia [20]), Europe (one each in Germany [37], Spain [33], Sweden [35], United Kingdom [34]), Latin America (two in Brazil [32,50]), and Africa (one in Ethiopia [48]). Sample size ranged from a minimum of 56 participants [35] to a maximum of 2498 [42], for an overall sample size of 10,771.

Medical students were the most investigated population, with 13 studies focusing only on undergraduate medical students [20,24,25,26,29,31,35,37,39,41,42,49]. Three studies focused on dental students and interns [22,30,40], two studies on pharmacy students [13,48], and two studies on nursing students [36,38]. There were three studies with mixed populations of healthcare students (i.e., students in nursing, medicine, dentistry, occupational therapy, speech therapy, and pharmacy in Cauduro et al. [32]; students in medicine, nursing, and midwifery in Nabilou et al. [43]; students in medicine and nursing in Yoshikawa et al. [50]). Two studies assessed mixed populations of medical students and residents [46,47]. A detailed overview of the study characteristics is provided in Supplementary File 6.

### 3.3. Overall Attitudes of Students and Young Health Professionals

**Figure 1:**
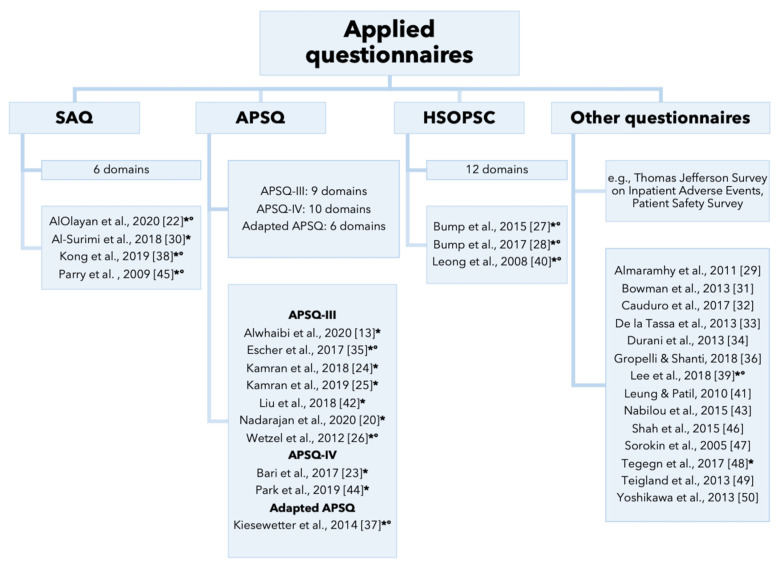
Different types of questionnaires used in the studies. Notes. * Use of questionnaire validated in original language; ° Use of questionnaire validated in language in which it was administered.

The primary studies administered different questionnaires to assess young health professionals’ attitudes towards patient safety culture (see Figure 1 and Supplementary File 7).

**Figure 2:**
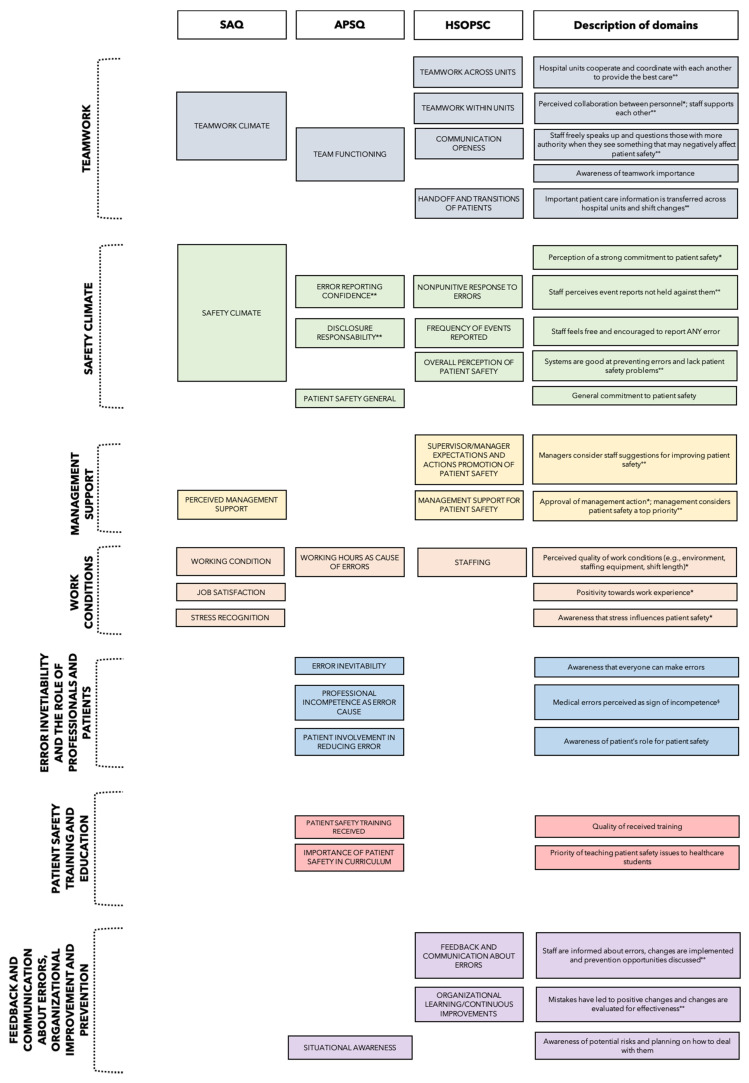
Overview of the domains of the most applied questionnaires, validated in their original language (SAQ, APSQ, HSOPSC). Notes. ** the dimension safety climate includes items regarding “error reporting confidence” and “disclosure responsibility”; § all items reversed (reversed dimension); * Adapted from Sexton et al. [51], °° Adapted from Sorra et al. [52].

Figure 2 gives a visual overview of the content-related similarities and differences between the individual domains of the most applied questionnaires, all of which validated in their original language (i.e., SAQ, APSQ, HSOPSC), and illustrates the structure of the narrative synthesis of the results (i.e., synthesis of results across domains rather than across tools).

#### 3.3.1. Teamwork

As regards the SAQ domain *teamwork climate,* acceptable mean scores were registered across studies, ranging from 61.18 [22] to 67.26 [38] but positive response rates reached only up to 60% (i.e., percentage of participants giving the desired/correct response which demonstrates a positive attitude towards patient safety). A detailed overview of the mean scores and percentages of positive answers per patient safety domain reported by studies administering SAQ, APSQ, and HSOPC is given in Supplementary File 8.

In four studies using the APSQ, the domain *team functioning* obtained the highest mean score [23,24,25,35]. Positive response rates of 94.6% [20] and 88.8% [26] were the highest and second highest percentages, respectively, at domain level in these studies. Regarding the articles administering the HSOPSQ, the domains *communication openness* and *handoffs and transitions of patients* received lower percentages (between 42% [40] and 55% [28] and between 38% [27] and 46% [40], respectively) than the domains *teamwork within units* (between 60% [27,28] and 74% [40]), and *teamwork across units* (between 56% [27] and 73% [28]).

In articles using other questionnaires than SAQ, APSQ, and HSOPSC, teamwork was considered particularly important in four instances [31,34,39,47].

#### 3.3.2. Safety Climate

While the mean score of the SAQ domain *safety climate* in the study by Parry et al. [45] reflected a very positive attitude (76.1), mean scores were generally lower in other studies (59.73 [22], 66.16 [38]). Kong et al. [38] and Al-Surimi et al. [30] found the lowest percentage of positive responses (30.7% and 40.7%, respectively) for this domain.

The positive response rate for the APSQ domain *error reporting confidence* was equally high in Liu et al. [42] (74.9%) and Nadarajan et al. [20] (76.3%), while Park et al. [44] and Wetzel et al. [26] showed numbers below 60%. In the latter study, it also represented the lowest percentage at domain level [26]. Low percentages of positive responses were also found for the HSOPSC domain *nonpunitive responses to errors* (between 35% [40] and 40% [27,28]). Moreover, 48% of respondents in Bowman et al. [31] and 39% in Gropelli and Shanti [36] reported that their mistakes were held against them. On the other hand, Almaramhy et al. [29] stated that 80.7% of respondents would not blame peers for their own mistake and 76% would support peers who make unintentional errors.

Regarding *disclosure responsibility*, two studies recorded the lowest mean scores for this APSQ domain [23,25], with positive response rates ranging between 56.90% [13] and 77.0% [44]. Similarly, the HSOPSC domain *frequency of adverse events reported* ranged only between 37% [40] and 58% [27].

The studies applying other questionnaires than the SAQ, APSQ, and HSOPSC offered conflicting results on attitudes towards error disclosure. While some articles recorded overwhelmingly positive responses [31,41,46,47], others indicated more negative attitudes towards error disclosure [29,32,33,43].

Positive responses to the HSOPSC domain *overall perception of patient safety* ranged between 55% [27,28] and 63% [40]. Similar to this domain is the APSQ domain *general patient safety* which was, however, only present in the two studies applying the APSQ-IV [23,44]. Only Park et al. [44] provided frequencies, reporting a high rate of positive responses of 74.4%.

Finally, a clear perception of the overall importance of patient safety emerged from the studies administering other instruments than SAQ, APSQ, and HSOPSC [29,41,46].

#### 3.3.3. Management Support

Mean scores for the SAQ domain *management support* were between 50.7 [30] and 70.4 [45] and relatively low percentages of positive responses between 44.8% [30] and 47.6% [22] were observed.

Relatively high percentages of positive response were found for the HSOPSC domain *supervisors’ expectations and actions promoting patient safety* (between 67% [40] and 80% [27,28]. However, percentages of positive responses of the domain *management support for patient safety* were lower (between 55% [27] and 66% [40].

#### 3.3.4. Work Conditions

Results for the SAQ domain *work conditions* were acceptable, with mean scores ranging from to 64.74 [22] to 75.6 [45]. However, percentages of positive responses varied greatly (35.9% reported by Kong et al. [38], 57% reported by Al Surimi et al. [30]).

The APSQ domain *working hours as cause of error* obtained one of the highest mean scores in three studies [24,25,35]. Similarly, the percentage of positive responses was overall high, reaching 89.5% in Nadarajan et al. [20]. The lowest frequency (73.4%) was reported by Wetzel et al. [26].

On the other hand, the HSOPSC domain *staffing* received somewhat lower percentages of positive responses, ranging from 54% [40] to 60% [27,28].

Closely linked to the perceived quality of work conditions are the SAQ domains *job satisfaction* and *stress recognition. Job satisfaction* reached the highest mean score in two studies (70.25 [22] and 77.5 [45]) and the highest rate of positive responses in two other studies (65.6% [30], 67.9% [22]).

Results for the domain *stress recognition* showed some variability across studies. In one study [38], it was the domain with the highest positive response rate (51.5%) and a positive mean score of 70.75, whereas in other studies [22,30,45] the mean scores were lower.

#### 3.3.5. Error Inevitability and the Role of Professionals and Patients

Three studies [35,37,44] reported high mean scores for the APSQ domain *error inevitability* (highest mean score in Kiesewetter et al. [37] and Park et al. [44], one of the highest in Escher et al. [35]). Percentages of positive responses for this domain ranged from 59.1% [13] to 92.8% in Wetzel et al. [26], representing the highest positively rated domain in this study.

Beliefs and perceptions about the inevitability of errors differed across the studies that used other questionnaires. In two studies, between 50% [29,33] and 61% of respondents [47] agreed that most clinical errors are preventable. On the contrary, in five other studies [32,41,43,46,50], between 36.7% [32] and 72% [41] stated that mistakes are inevitable in healthcare.

While the APSQ domain *professional incompetence as error cause* obtained the lowest mean score in three studies [13,20,24], it achieved one of the highest mean scores in Escher et al. [35]. Across studies, the positive response rate ranged from only 23.7% in the study by Alwhaibi et al. [13] (lowest percentage by domain in the study) to 70.0% in Nadarajan et al. [20].

Similarly, in the studies using other questionnaires, results were inconsistent regarding the perception that competent professionals do not make errors, with percentages ranging from 0% [34] to 82% [29].

The APSQ domain *patient involvement in reducing errors* received the lowest mean score across domains by Escher et al. [35] but overall obtained percentage responses between 61.4% [44] to 87.4% [26].

Studies using other questionnaires than SAQ, APSQ, and HSOPSC reported that between 44.7% [29] and 61.0% [47] of participants see patients as playing a role in preventing or causing adverse events.

#### 3.3.6. Patient Safety Training and Education

The overall positive response rate of the APSQ domain *patient training received* ranged from 59.6% [13] to 85.2% in Nadarajan et al. [20]. Liu et al. [42] reported that it was the most positively rated domain. On the other hand, in the study by Kiesewetter et al. [37], the mean score of 3.76 represented the lowest value at the domain level. The range of positive responses to the APSQ domain *importance of patient safety in curriculum* was similar, with percentages between 55.8% [44] and 80.1% [26].

Across studies using other questionnaires, more than two thirds of respondents expressed interest in learning about different aspects of patient safety and highlighted the importance of patient safety education in healthcare schools and continuous training for healthcare staff [29,31,41,43,46,47,48].

#### 3.3.7. Feedback and Communication about Errors, Organizational Improvement, and Prevention

A low rate of endorsement was found for the HSOPSC domain *feedback and communications about errors*, with percentages ranging from 38% [40] to 53% [28]. Similarly, in the study by Lee et al. [39], only 33% agreed that they received appropriate feedback about their performance and only 34.5% that the clinical culture made it easy to learn from the errors of others. On the contrary, Cauduro et al. [32] using an ad hoc questionnaire, reported that 90.1% of respondents agreed that when the error occurs, all those involved should discuss the event.

The HSOPSC domains *organizational learning/continuous improvements* also received only positive response rates of up to 65% [28]. *Situational awareness*, namely awareness of potential risks and planning on how to deal with them, was only assessed by two studies [23,44] applying the APSQ-IV. A great number of participants considered it important for patient safety to understand the roles and responsibilities of every member of the team (88%) and to plan together to deal with potential problems (83.3%) [44]. These aspects received also positive scores in the study by Bari et al. [23]. However, 72% of respondents in the study by Park et al. [44] got the negatively worded item “Being on the look-out for potential risks can be detrimental for patient safety” wrong.

Regarding studies using other questionnaires than the SAQ, APSQ, and HSOPSC, a systemic analysis of the facts to implement preventive measures was considered as important by 86.4% of respondents [32]. Policies and procedures were seen as good at preventing error by almost all participants of Gropelli and Shanti [36] (93%). In terms of strategies to prevent errors, over 75% of respondents believed that the most effective strategy to prevent errors is to work harder and be more careful [32,41,48,50].

#### 3.3.8. Differences in Patient Safety Attitudes between Subgroups

A structured synthesis of statistically significant group differences in patient safety attitudes psychological and psychosomatic reactions is provided in Table 1.

#### 3.3.9. Differences across Years of Study

The 16 studies investigating differences across years of study [20,22,23,25,27,30,33,34,36,37,38,42,43,46,47,48] yielded heterogenous results. Five indicated that more advanced students showed a more positive attitude towards different aspects of patient safety (e.g., teamwork climate, perception of management error reporting, disclosure responsibility) [22,30,33,46,47] (see Table 1). For instance, AlOlayan et al. [22] found a significant increase in the mean scores for several patient safety domains from the fourth year to the internship (*p* < 0.01 for all). By contrast, three articles [23,36,37] showed more positive attitudes in younger students. For example, Kiesewetter et al. [37] stated that final year medical students showed significantly lower values of the APSQ scale *error reporting confidence*, than students between the first and fifth year (*p* < 0.000).

Three studies [20,34,42] reported heterogenous results for students from different years and five studies [25,27,38,43,48] did not find any significant association between patient safety attitudes and years of study.

#### 3.3.10. Differences between Genders

Five studies observed significant differences between genders [13,20,22,35,43], with women tendentially showing a more positive attitude towards patient safety (see Table 1).

Alwhaibi et al. [13] reported a more positive attitude of women in nearly all APSQ domains, namely *patient safety training received, error reporting confidence, working hours as error cause, error inevitability, team functioning*, and *patient involvement in reducing errors* (all *p* < 0.05). Male students had a more positive attitude only in the domain *professional incompetence as error* (*p* < 0.05) [13].

On the contrary, in the study by Nadarajan et al. [20], women scored higher on *professional incompetence* (*p* = 0.012), whereas men scored higher in *error reporting confidence* (*p* = 0.002). In the study by AlOlayan and colleagues [22], female students scored higher in the domain *stress recognition* (*p* = 0.004). Similarly, women showed higher scores in the APSQ sub-scores *disclosure responsibility* (*p* < 0.001) and *team functioning* (*p* = 0.029) [30]. However, in the study by Nabilou et al. [43], men were more interest in patient safety education (*p* = 0.001).

Four of the overall nine studies comparing gender did not detect any significant differences in attitudes [24,25,38,48].

#### 3.3.11. Differences across Specialties

Four studies reported statistically significant differences between different specialties [30,31,34,43] (see Table 1).

In the study by Al-Surimi et al. [30], dentistry students were significantly more likely (*p* < 0.001) to have positive perceptions in all six domains of the SAQ compared with dental hygiene students. In the study by Durani et al. [34], significantly fewer surgical than medical trainees gave a desired response to several individual items, including “Medical error is a sign of incompetence” (*p* < 0.001) and “It is only important to disclose errors to patients if they have resulted in harm” (*p* < 0.001). Similarly, Bowman et al. [31] showed that surgical students rated the domain *teamwork significantly* less positive than internal medical students (*p* < 0.05). Nabilou et al. [40] stated that nursing and midwifery students were more interested in learning patient safety topics and obtained higher scores on perceptions of patient safety than medical students (*p* = 0.0017 and *p* = 0.001, respectively).

## 4. Discussion

To our knowledge, this is the first systematic review of patient safety attitudes among healthcare profession students and young professionals. Data heterogeneity across studies was moderate to high, reflecting the changing nature of safety climate (i.e., a snapshot of safety perception at a specific point in time) [9], as well as the great variety of types and versions of applied questionnaires. Nevertheless, it was still possible to identify certain trends regarding students and young professionals’ attitudes towards safety.

One important finding was the overall high rating given to teamwork across studies. In general, young professionals recognized the importance of effective teamwork for patient safety and particularly for error reduction. They were also able to identify potentially critical aspects of teamwork, such as communicating problems and managing disagreements. It is important to bear in mind that context influences how teams function [53]. Specifically, different hospital and clinical units within hospitals may have their own perceptions towards teamwork. These subcultures or microclimates influence how team members interact with each other. Here also the “hidden curriculum” comes into play [54,55], defined as “learning that occurs by means of informal interactions among students, faculty, and others and/or learning that occurs through organizational, structural, and cultural influences intrinsic to training institutions” (p. 1709) [54]. This informal learning that occurs in clinical practice and might be influenced by geographic factors [56,57] can have a fundamental role in shaping one’s attitudes towards patient safety and care [55,58]. To foster the development of a positive safety culture, it is essential that the hidden curriculum corresponds as closely as possible to the contents of the planned and formal training (i.e., explicit curriculum). In this way, the hidden curriculum provides positive reinforcement for positive safety attitudes that may ultimately coalesce into a culture of safety. On the other hand, subcultures ignore or oppose the contents of the formal training and positive safety attitudes may be opposed with a negative impact on training efficacy.

It is interesting to note that the domains *perceived management support* (SAQ) and *management support for patient safety* (HSOPSC) obtained lower scores than another dimension related to supervision, namely *supervisors’ expectations and actions promoting patient safety* (HSOPSC)*,* that obtained overall higher scores. This finding might be attributed in part to the discrepancy between the more conservative, hidden curriculum and the more progressive, formal patient safety education [12,59].

A striking observation was the overall relatively low scores of the domains *safety climate* (SAQ), *disclosure responsibility* (APSQ), and *frequency of adverse events reported* (HSOPS). These findings point to a still prevalent culture of under-reporting of adverse events and medical errors. This is pronounced in disclosing to patients and their families, due in part to fear of consequences [4,60,61,62,63]. Closely related to this finding was the discouragingly low ratings of the HSOPSC domains *feedback and communication about errors* and *non-punitive response to errors* and of related areas in ad hoc questionnaires. In fact, a good feedback system remains the essential element to create a positive learning environment and to promote reporting of incidents. To be effective and induce change, reporting must be followed by adequate actions and management (e.g., well established feedback mechanisms, implementation of appropriate corrective actions, disseminations of results throughout the organization to promote learning processes) [64]. Although organizations are increasingly equipped with procedures for reporting events, structured systems for managing the event tend to be less well developed [65]. In line with this, the domain *organizational learning/continuous improvement* (HOSPSC) did not receive particularly positive scores. By contrast, evaluations of the dimension *error inevitability* were generally positive, thus indicating that the majority of students were oriented towards a blame-free culture and adopted a systemic approach to understanding error [66]. However, misconceptions about causation (e.g., medical errors as sign of incompetence) persisted in some studies.

With regard to subgroup comparisons, our formal narrative synthesis yielded heterogenous findings on patient safety attitudes over the course of training. Some studies indicated that with increasing years of experience, students seem to become more aware of the importance of teamwork, error reporting and disclosure responsibility. However, they also seem to lose confidence in their ability to report these errors, as reported by Kiesewetter et al. [37]. This might be another effect of the hidden curriculum to which students are progressively exposed as they spend more time in real world settings.

Women also seemed to hold more positive patient safety attitudes. Research on this aspect is still lacking, and we can only speculate as to the reasons for this observation. It might be related to differences in clinical practice patterns and quality of care between women and men [67]. Indeed, several authors indicated that women might be more apt to follow clinical instructions, and to deliver more patient-centered and preventive care [67,68,69,70,71,72,73]. A recent study by Tsugawa et al. [67] even suggested a lower mortality and fewer readmissions among patients cared for by female internists than by their male colleagues.

Of the four studies comparing different specialties [30,31,34,43], only two [31,34] focused on the same specialty (e.g., surgery vs medicine), demonstrating lower awareness of patient safety topics among surgical trainees/students than medical trainees/students. This result may be linked to the greater importance of skills and technical procedures in surgery, or prevailing attitudes of senior colleagues.

### 4.1. Recommendations for Medical Education and Clinical Practice

The findings of this study have several practical implications for different, intertwined levels of the healthcare system (i.e., healthcare provider, organizational, institutional level).

Health care organizations should develop a learning culture, characterized by open communication, transparency, and cooperation. Education on patient safety should be incorporated in healthcare workers’ training from early on [12,74]. As Kiesewetter et al. [37] emphasized, “the question should no longer be if, but how medical faculties should implement curricula regarding patient safety and medical error” (p. 509). This position is supported by our findings, which found a great interest in patient safety education among students and trainees. Our findings suggest that healthcare curricula should focus more on safety climate, error communication, disclosure responsibility, and misconceptions about error causes. These elements should be reinforced and continued in clinical practice in team-based learning sessions, quality improvement activities, debriefings, and clinical simulations [26,36,49,75,76].

Healthcare institutions and health profession schools should also address the impact of the hidden curriculum on students and young professionals [12,39,59]. Continued efforts are necessary to understand and influence the traditional, informal curriculum that still too often reflects a punitive culture. It will be important to better align it to the more forward-thinking explicit curriculum that is increasingly taught. Encouraging role-modelling by senior colleagues (e.g., thoughtful error disclosure to patients and their family members) could help in this endeavor [12,31,38]. In the long run, improving safety culture and teamwork climate might contribute to decreasing patient harm and even hospital mortality [12,77].

Promoting a positive safety culture in education and clinical practice is also important because of the relationship between burnout and patient safety. Burnout, which is highly prevalent among all health professions, is also associated with lower levels of patient safety [78]. On the other hand, establishing a strong safety culture can have a positive impact on healthcare workers’ burnout [79]. This bidirectional relationship underlines how insufficient attention to safety culture may trigger a vicious cycle, with a poor safety culture leading to burnout, and in turn to decreased patient safety. In light of the current COVID-19 pandemic and its immense impact on healthcare workers’ mental health [80], the prevention of burnout among healthcare staff should now play an even greater role.

### 4.2. Limitations of Our Study

This study has some potential shortcomings. First, several primary studies did not use validated questionnaires or did not clearly state whether a validated translation had been used. Moreover, the included studies showed great heterogeneity in many regards, such as applied type and version of questionnaire (e.g., different lengths of Likert scales, items differing in their wording), registration, and presentation of data. For instance, some studies did not provide mean scores or percentages, rendering difficult the synthesis and comparison of findings across studies and with benchmarking data. The primary studies were also heterogeneous with respect to the examined study population. Participants were from different geographical backgrounds, healthcare professions, and medical specialties, as well as years of study. However, we only presented subgroup comparisons regarding differences across years of study, specialty, and gender since preliminary analyses regarding the geographic location did not show any clear differences or trends. Other comparisons of interest, such as differences in attitudes between students with low and high workload, were not conducted often enough to make subgroup analyses possible. Further, some biases, such as social desirability bias, recall bias, and non-response bias may have influenced the results of the included studies and been reflected in turn in our findings. Another limitation is that we only focused on patient safety attitudes and did not take into account other aspects of patient safety culture, such as knowledge, skills, and behaviors. Although such an expanded focus would have produced additional information/evidence, the large number of eligible, heterogeneous studies was beyond the scope of this paper. Moreover, the lack of a controlled vocabulary may have decreased the precision of our search to a certain extent [81]. Finally, an intrinsic limitation of our applied methodology is that it can be prone to subjective valuation. However, we tried to counter this by involving two reviewers at each of the methodologic steps.

### 4.3. Future Research Directions

We call for more research on the current topic, particularly in Italy and other European countries. More studies are also needed to better understand how students and early career healthcare providers from different (sub)specialties differ in their patient safety attitudes. This evidence could help to tailor patient safety training to their specific needs. Further work is also required to examine the potential role of geographic factors on health profession students’ attitudes towards patient safety. More data are also needed to determine why women appear to have more positive patient safety attitudes than men and if it this tendency is connected to their practice patterns. Moreover, multi-site, longitudinal studies following healthcare profession students during undergraduate and specialty training could examine the development of students’ safety attitudes over time and links to influencing factors, such as the experience of medical errors and patient safety education. Monitoring safety culture over the course of training might also help to prevent young professionals from burnout and help them to maintain high levels of motivation and job satisfaction. Additional studies comparing students’ and young professionals’ attitudes with those of senior professionals and supervisors could shed more light on the differences between formal and hidden curricula.

Finally, a natural progression of this study would be to systematically review and synthesize the literature on the patient safety knowledge and behavior among aspiring healthcare providers to get a more complete picture of their approach towards patient safety.

## 5. Conclusions

Based on data reported by 10,771 health care trainees, our study underlines that students and early stage professionals showed more positive patient safety attitudes in some areas, such as teamwork climate, error inevitability, received patient safety training, and importance of patient safety in the curriculum. However, they also held more negative perceptions in other domains, such as management support, safety climate, error communication, disclosure responsibility, and professional incompetence as a cause of errors. Women and individuals with more years of training tended to exhibit more positive attitudes toward patient safety. Taken together, our findings have implications for future practice. Creating a learning culture by incorporating patient safety education in curricula of future healthcare providers and by promoting their direct, active involvement in patient safety procedures should be a priority for policy makers, healthcare managers, and clinicians. Considering the influence of the hidden curriculum on aspiring health care professionals, health care organizations should also ensure that the hidden curriculum better mirrors the values of the explicit curriculum. This will be necessary to create the basis for developing a strong, positive safety culture and promoting the delivery of high quality of care.

## Figures and Tables

**Table 1:** Significant differences in patient safety attitudes between subgroups.

**DIFFERENCES ACROSS YEARS OF STUDIES**		
	**Less Advanced Students**	**More Advanced Students**
AlOlayan et al. [22]		SAQ domains *Teamwork climate, Safety climate,* *Perception of management, Work condition* (*p* < 0.01 for all)
Al-Surimi et al. [30]		SAQ domain *Teamwork climate* (*p* = 0.001)
Bari et al., 2017 [25]	Positively worded questions on patient safety attitudes (*p* = 0.006)	
De la Tassa et al. [33]		Perception of importance of improvements in techniques and procedures and of involvement in group for patient safety improvement (*p* < 0.05 for all)
Durani et al. [34]	Individual items *“Medical error is a sign of incompetence”* (*p* < 0.001), “*It is only important to disclose errors to patients if they have resulted in harm”* (*p* = 0.008)	Individual items “*Management is more interested in meeting performance targets than focusing on patient safety issues”* (*p* < 0.001), *“My suggestions about patient safety would be acted upon if I expressed them to management”* (*p* < 0.001), *“I know the proper channels to direct questions regarding patient safety”* (*p* < 0.001), *“The senior managers in my hospital listen to me and care about my patient safety concerns”* (*p* < 0.001), *“The senior doctors in my department listen to me and care about my patient safety concerns”* (*p* = 0.002), *“I would feel safe here being treated as an inpatient”* (*p* = 0.004)
Gropelli and Shanti [36]	Individual items *“As a student, I have a safety focus for my patient”* (*p* < 0.020), “*My patient has a safety focus for my shift”* (*p* < 0.028), *“My clinical instructor focuses on safety issues”* (*p* < 0.039), *“Students are informed about errors that happened during the semester”* (*p* < 0.021)	
Kiesewetter et al. [37]	APSQ scale *Error reporting confidence* (*p* < 0.000)	
Liu et al. [42]	APSQ domains *Working hours as an error cause* and *Teamwork* (*p* < 0.05)	APSQ domain *Error inevitability* (*p* < 0.05)
Nadarajan et al. [20]	APSQ domain *Disclosure responsibility* (*p* = 0.002)	APSQ domain *Error reporting confidence* (*p* = 0.001), *Professional incompetence* (*p* < 0.001)
Shah et al. [46]		Individual items *“There is no need to report a near miss event”* (*p* = 0.01), *“Only physicians can determine the cause of medical errors”* (*p* < 0.001), “*Most errors are not related to physicians”* (*p* = 0.04)
Sorokin et al. [47]		*Work efficiency* (*Reduction of adverse events by establishing 80-h workweek*) (*p* = 0.03) *Comfort in disclosure discussions* (*p* < 0.01)
**DIFFERENCES BETWEEN GENDERS**		
	**Women**	**Men**
AlOlayan et al. [22]	SAQ domain *Stress recognition* (*p* = 0.004)	
Alwhaibi et al. [13]	APSQ domains *Patient safety training received*, *Error reporting confidence*, *Working hours as error cause, Error inevitability*, *Team functioning*, *Patient involvement in reducing errors* (*p* < 0.05)	APSQ domain *Professional incompetence as error* (*p* < 0.05)
Escher et al. [35]	APSQ domains *Disclosure responsibility* (*p* < 0.001)*; Team functioning* (*p* = 0.029)	
Nadarajan et al. [20]	APSQ domain *Professional incompetence as error* (*p* = 0.012)	APSQ domain *Error reporting confidence* (*p* = 0.002)
Nabilou et al. [40]		*Interest in patient safety education* (*p* = 0.001)
**DIFFERENCES ACROSS SPECIALTIES**		
	**Dentistry Students**	**Dental Hygiene Students**
Al-Surimi et al. [30]	SAQ domains *Teamwork climate, Safety climate, Job satisfaction, Stress recognition, Perceived management support, Working conditions* (*p* < 0.04)	
	**Surgical Students/Trainees**	**Medical Students/Trainees**
Bowman et al. [31]		*Teamwork* (*p* < 0.05)
Durani et al. [34]		Individual items “*The number of hours doctors work increases the likelihood of making errors”* (*p* = 0.035), “*Medical error is a sign of incompetence*” (*p* < 0.00), “*Learning about patient safety is not as important as learning other more skill-based aspects of being a doctor*” (*p* < 0.001), “*It is only important to disclose errors to patients if they have resulted in harm”,* (*p* < 0.00)
	**Nursing/Midwifery Students**	**Medical Students**
Nabilou et al. [43]	*Interest in patient safety education* (*p* = 0.0017) *Attitude towards patient safety* (*p* = 0.001)	

## Data Availability

The data presented in this study are available on request from the corresponding author.

## Supplementary Materials

The following are available online at https://www.mdpi.com/article/10.3390/ijerph18147524/s1. Supplementary File 1: Search strategy and retrieved records from each electronic database; Supplementary File 2: Additional Searches; Supplementary File 3: List of excluded studies; Supplementary File 4: PRISMA Flow Diagram; Supplementary File 5: Risk of bias assessment of included studies; Supplementary File 6: Characteristics of included studies; Supplementary File 7: Characteristics of administered questionnaires; Supplementary File 8: Mean scores and percentages of positive answers per patient safety domain reported by studies applying SAQ, APSQ, and HSOPSC.
