# Supplementary material for: Patient Safety in the Eyes of Aspiring Healthcare Professionals: A Systematic Review of Their Attitudes
Source: Int J Environ Res Public Health. 2021 Jul 15;18(14):7524. doi: 10.3390/ijerph18147524 (PMC8306767; doi:10.3390/ijerph18147524)
Supplement: Supplementary file 1 [file ijerph-18-07524-s001.zip › ijerph-1249972-supplementary.pdf]

Supplementary File 1. Search strategies and retrieved records from each electronic database

1. PubMed

   Date of search: 02/18/2020

   Search strategy:

   #1 (patient safety OR patient safety attitude OR safety climate OR patient safety competence OR patient safety culture OR patient safety values OR patient safety behavior)
   #2 (students OR junior doctors OR newly graduates OR newly registered OR postgraduate trainees OR resident trainees)

   #1 AND #2

   N findings: 2798

2. Web of Science (core collection) search strategy

   Date of search: 02/19/2020

   Search Strategy:

   #1 (patient safety OR patient safety attitude OR safety climate OR patient safety competence OR patient safety culture OR patient safety values OR patient safety behavior)
   #2 (students OR junior doctors OR newly graduates OR newly registered OR postgraduate trainees OR resident trainees)

   #1 AND #2

   N findings: 4305

3. Scopus

   Date of search: 02/20/2020

   Search Strategy:

   (patient safety OR patient safety attitude OR safety climate OR patient safety competence OR patient safety culture OR patient safety values OR patient safety behavior) AND (students OR junior doctors OR newly graduates OR newly registered OR postgraduate trainees OR resident trainees)

   N findings: 300

4. PsycINFO

   Date of search: 03/20/2020

   Search strategy:

   #1 (patient safety OR patient safety attitude OR safety climate OR patient safety competence OR patient safety culture OR patient safety values OR patient safety behavior)
   #2 (students OR junior doctors OR newly graduates OR newly registered OR postgraduate trainees OR resident trainees)

   #1 AND #2

   N findings: 1494

Supplementary File 2. Additional searches

Open Grey Database

- Search conducted on September 28, 2020
- Search strategy: (patient safety OR patient safety attitude OR safety climate OR patient safety competence OR patient safety culture OR patient safety values OR patient safety behaviour) AND (students OR junior doctors OR newly graduates OR newly registered OR postgraduate trainees OR resident trainees)
- Records: n=6

Grey Literature Report

- Search conducted on September 28, 2020
- Search strategy: (patient safety OR patient safety attitude OR safety climate OR patient safety competence OR patient safety culture OR patient safety values OR patient safety behaviour) AND (students OR junior doctors OR newly graduates OR newly registered OR postgraduate trainees OR resident trainees)
- Records: n=0

Weekly email search alerts

- We screened automatic weekly email search alerts on Pubmed from February 18, 2020 until October 18, 2020.

Reviews

- Abbas MR, Quince TA, Wood DF, Benson JA. Attitudes of medical students to medical leadership and management: a systematic review to inform curriculum development. BMC Med Educ. 2011 Nov 14;11:93. doi: 10.1186/1472-6920-11-93. PMID: 22082174; PMCID: PMC3247079.
- Boonyasai RT, Windish DM, Chakraborti C, Feldman LS, Rubin HR, Bass EB. Effectiveness of teaching quality improvement to clinicians: a systematic review. JAMA. 2007 Sep 5;298(9):1023-37. doi: 10.1001/jama.298.9.1023. PMID: 17785647.
- Kirkman MA, Sevdalis N, Arora S, Baker P, Vincent C, Ahmed M. The outcomes of recent patient safety education interventions for trainee physicians and medical students: a systematic review. BMJ Open. 2015 May 20;5(5):e007705. doi: 10.1136/bmjopen-2015-007705. PMID: 25995240; PMCID: PMC4442206.
- Monrouxe LV, Grundy L, Mann M, John Z, Panagoulas E, Bullock A, Mattick K. How prepared are UK medical graduates for practice? A rapid review of the literature 2009-2014. BMJ Open. 2017 Jan 13;7(1):e013656. doi: 10.1136/bmjopen-2016-013656. PMID: 28087554; PMCID: PMC5253586.
- Nie Y, Li L, Duan Y, Chen P, Barraclough BH, Zhang M, Li J. Patient safety education for undergraduate medical students: a systematic review. BMC Med Educ. 2011 Jun 14;11:33. doi: 10.1186/1472-6920-11-33. PMID: 21669007; PMCID: PMC3128569.
- Willmott J, Mould J. Health professionals' perception of patient safety culture in acute hospitals: an integrative review. Aust Health Rev. 2018 Aug;42(4):387-394. doi: 10.1071/AH16274. PMID: 28554000.

Supplementary File 3. List of excluded studies.

	Studies	Reason of Exclusion
1	Abu-El-Noor, N.I.; Abu-El-Noor, M.K.; Abuowda, Y.Z.; Alfaqawi, M.; Böttcher, B. Patient safety culture among nurses working in Palestinian governmental hospital: a pathway to a new policy. *BMC. Health. Serv. Res.* **2019**,*19*, 550.	Mixed population
2	Aboumatar, H.J.; Thompson, D.; Wu, A.; Dawson, P.; Colbert, J.; Marsteller, J.; Kent, P.; Lubomski, L.H.; Paine, L.; Pronovost, Peter. Republished: development and evaluation of a 3-day patient safety curriculum to advance knowledge, self-efficacy and system thinking among medical students. *Postgraduate Medical Journal.* **2012**, *88*, 545–551.	Mismatch with inclusion criteria
3	Ahmed, M.; Arora, S.; Carley, S.; Sevdalis, N.; Neale, G. Junior doctors' reflections on patient safety. *Postgraduate Medical Journal*. **2012**, *88*, 125–129.	Mismatch with inclusion criteria
4	Aldossary, S.A. Patient safety attitudes of clinical Pharmacy Students attending undergraduate program in King Faisal University. *Pak. J. Pharm. Sci*. **2019**, *32*, 471–475.	Mismatch with inclusion criteria
5	Alquwez, N.; Cruz, J.P.; Alshammari, F.; Felemban, E.M.; Almazan, J.U.; Tumala, R.B.; Alabdulaziz, H.M.; Alsolami, F.; Silang, J.P.B.T.; Tork, H.M.M. A multi-university assessment of patient safety competence during clinical training among baccalaureate nursing students: A cross-sectional study. *J. Clin. Nurs.* **2019**, *28*, 1771–1781.	Mismatch with inclusion criteria
6	Al Sweleh, F.S.; Al Saedan, A.M.; Al Dayel, O.A. Patient safety culture perceptions in the college of dentistry. *Medicine (Baltimore)*. **2018**, *97*, e9570.	Mismatch with inclusion criteria
7	Anderson, E.; Thorpe, L.; Heney, D.; Petersen, S. Medical students benefit from learning about patient safety in an interprofessional team. *Medical Education.* **2009**, *43*, 542–552.	Mismatch with inclusion criteria
8	Attree, M.; Cooke, H.; Wakefield, A. Patient safety in an English pre-registration nursing curriculum. *Nurse. Educ. Pract*. **2008**, *8*, 239-48, doi:10.1016/j.nepr.2007.09.003.	Mismatch with inclusion criteria
9	Ayub, A.; Khan, R.A. Learning to cure with care: awareness of faculty and medical students about student's roles related to patient safety. *JPMA*. **2018,** *68*, 1350–1357.	Mismatch with inclusion criteria
10	Barratt, R.; Wyer, M.; Hor, S.Y.; Gilbert, G.L. Medical interns' reflections on their training in use of personal protective equipment. *BMC. Med. Educ*. **2020**, *23*, 328.	Mismatch with inclusion criteria
11	Beekman, M.; Emani, V.K.; Wolford, R.; Hanson, K.; Wickham, G.; Aiyer, M. Patient Safety Morning Report: Innovation in Teaching Core Patient Safety Principles to Third-Year Medical Students. *J. Med. Educ. Curric. Dev*. **2019**, *6*, 2382120519842539, doi:10.1177/2382120519842539.	Mismatch with inclusion criteria
12	Blasiak, R.C.; Stokes, C.L.; Meyerhoff, K.L.; Hines, R.E.; Wilson, L.A.; Viera, A.J. A cross-sectional study of medical students' knowledge of patient safety and quality improvement. *N. C. Med. J.* **2014,** *75*, 15–20.	Mismatch with inclusion criteria
13	Bottcher, B.; Abu-El-Noor, N.; Abuowda, Y.; Alfaqawi, M.; Alaloul, E.; El-Hout, S.; Al-Najjar, I.; Abu-El-Noor, M. Attitudes of doctors and nurses to patient safety and errors in medical practice in the Gaza-Strip: a cross-sectional study. *BMJ. Open*. **2019**, *9*, e026788.	Mixed population
14	Bradley, S.M.; Chang, D.; Fallar, R.; Karani, R. A patient safety and transitions of care curriculum for third-year medical students. *Gerontol. Geriatr. Educ*. **2015**, *36*, 45–57.	Mismatch with inclusion criteria
15	Bressan, V.; Stevanin, S.; Bulfone, G.; Zanini, A.; Dante, A.; Palese, A. Measuring patient safety knowledge and competences as perceived by nursing students: An Italian validation study *Nurse. Educ. Pract.* **2016**, *16*, 209–216.	Mismatch with inclusion criteria (validation study)
16	Carruthers, S.; Lawton, R.; Sandars, J.; Howe, A.; Perry, M. Attitudes to patient safety amongst medical students and tutors: Developing a reliable and valid measure. *Med. Teach.* **2009**, *31*, e370–6.	Mismatch with inclusion criteria (validation study)
17	Castello, M.; Ferrara, P.; Destrebecq, A.; Terzoni, S. The perception of clinical risk among students of different health professions: a multicentre study. *Br. J. Nurs*. **2019**, *28*, 193–197.	Mismatch with inclusion criteria
18	Chen, L.; Huang, F.; Yuan, X.; Song, J.; Chen, L. An Assessment of the Reliability and Factorial Validity of the Chinese Version of the Health Professional Education in Patient	Mismatch with inclusion criteria

	Safety Survey (H-PEPSS). *Front. Psychol*. **2019**, *10*, 2183, doi:10.3389/fpsyg.2019.02183.	(validation study)
19	Cohen, S.P.; Pelletier, J.H.; Ladd, J.M.; Feeney, C.; Parente, V.; Shaikh, S.K. Success of a Resident-Led Safety Council: A Model for Satisfying CLER Pathways to Excellence Patient Safety Goals. *J. Grad. Med. Educ.* **2019**,*11*, 226–230.	Wrong focus
20	Colet, P.C.; Cruz, J.P.; Cruz, C.P.; Al-Otaibi, J.; Qubeilat, H.; Alquwez, N. Patient Safety Competence of Nursing Students in Saudi Arabia: A Self-Reported Survey. *Int. J. Health. Sci. (Qassim)* 2015, *9*, 418–426.	Mismatch with inclusion criteria
21	Cooper, E. From the school of nursing quality and safety officer: nursing students' use of safety reporting tools and their perception of safety issues in clinical settings. *J. Prof. Nurs*. **2013**, *29*, 109–116.	Mismatch with inclusion criteria
22	Cox, K.R.; Scott, S.D.; Hall, L.W.; Aud, M.A.; Headrick, L.A.; Madsen, R. Uncovering differences among health professions trainees exposed to an interprofessional patient safety curriculum. *Qual. Manag. Health. Care.* **2009**,*18*, 182–193.	Mismatch with inclusion criteria
23	Cresswell, K.; Howe, A.; Steven, A.; Smith, P.; Ashcroft, D.; Fairhurst, K.; Bradley, F.; Magnusson, C.; McArthur, M.; Pearson, P.; Sheikh, A. Patient safety in healthcare preregistration educational curricula: multiple case study-based investigations of eight medicine, nursing, pharmacy and physiotherapy university courses. *BMJ. Qual. Saf*. **2013**, *22*, 843–854.	Mismatch with inclusion criteria
24	Dankbaar, M.E.; Richters, O.; Kalkman, C.J.; Prins, G.; Ten Cate, O.T.; van Merrienboer, J.J.; Schuit, S.C. Comparative effectiveness of a serious game and an e-module to support patient safety knowledge and awareness. *BMC. Med. Educ*. **2017**,*17*, 30.	Mismatch with inclusion criteria
25	Danko, C.L. The Effect of an Undergraduate Perioperative Nursing Course on afety Knowledge. *AORN. Journal.* **2019**, *109*, 346–354.	Mismatch with inclusion criteria
26	Daud-Gallotti, R.M.; Morinaga, C.V.; Arlindo-Rodrigues, M.; Velasco, I.T.; Martins, M.A.; Tiberio, I.C. A new method for the assessment of patient safety competencies during a medical school clerkship using an objective structured clinical examination. *Clinics. (Sao Paulo, Brazil)* **2011**, *66*, 1209–1215.	Mismatch with inclusion criteria
27	Debourgh, G.A. Synergy for patient safety and quality: academic and service partnerships to promote effective nurse education and clinical practice. *J. Prof. Nurs.* **2012**, *28*, 48–61.	Mismatch with inclusion criteria
28	de Feijter, J.M.; de Grave, W.S.; Dornan, T.; Koopmas, R.P.; Scherpbier, A.J.J.A. Students' perceptions of patient safety during the transition from undergraduate to postgraduate training: an activity theory analysis. *Adv. Health. Sci. Educ. Theory. Pract.* **2011**,*16*, 347–358.	Mismatch with inclusion criteria
29	de Feijter, J.M.; de Grave, W.S.; Dornan, T.; Koopmas, R.P.; Scherpbier, A.J.J.A. Reflective learning in a patient safety course for final-year medical students. *Med. Teach*. **2012**, *34*, 946–954.	Mismatch with inclusion criteria (focus group)
30	Digby, G.C. Improving a patient safety curriculum for medical students. *Med. Educ*. **2020**, *54*, 1045–1046.	Mismatch with inclusion criteria
31	Doering, A.; Stueven, J.; Kalishman, S.; Wayne, S.; Sklar, D. Can medical students identify problems in patient safety? *Am. J. Med. Qual*. **2015**, *30*, 395–396.	Mismatch with inclusion criteria
32	Doyle, P.; VanDenKerkhof, E.G.; Edge, D.S.; Ginsburg, L.; Goldstein, D.H. Self-reported patient safety competence among Canadian medical students and postgraduate trainees: a cross-sectional survey. *BMJ. Qual. Saf*. **2015**, *24*, 135–141.	Mismatch with inclusion criteria
33	Dudas, R.A.; Bundy, D.G.; Miller, M.R.; Barone, M. Can teaching medical students to investigate medication errors change their attitudes towards patient safety? *BMJ. Qual. Saf.* **2011**, *20*, 319–325.	Mismatch with inclusion criteria
34	Duhn, L.; Karp, S.; Oni, O.; Edge, D.; Ginsburg, L.; VanDenKerkhof, E. Perspectives on patient safety among undergraduate nursing students. *J. Nurs. Educ.* **2012**, *51*, 526–531.	Mismatch with inclusion criteria
35	Dumenco, L.; Monteiro, K.; George, P.; McNicoll, L.; Warrier, S.; Dollase, R. An Interactive Quality Improvement and Patient Safety Workshop for First-Year Medical Students. *MedEdPORTAL.* **2018**, *14*, 10734.	Mismatch with inclusion criteria
36	Dumenco, L.; Monteiro, K.; George, P.; Rougas, S.; Dollase, R. Outcomes of a Longitudinal Quality Improvement and Patient Safety Preclerkship Curriculum. *Acad. Med*. **2019**, *94*, 1980–1987.	Mismatch with inclusion criteria

37	Eisenberg, M.A.; Nagler, J.; Moses, J.; Paul, R.; Hudgins, J. Development of a Longitudinal Quality and Safety Curriculum for Pediatric Emergency Medicine Fellows. *Clin. Pediatr. Emerg. Med.* **2017**, *18*, 130–137.	Mismatch with inclusion criteria
38	Eltony, S.A.; El-Sayed, N.H.; El-Araby, S.E.S.; Kassab, S.E. Implementation and evaluation of a patient safety course in a problem-based learning program. *Educ. Health. (Abingdon)* **2017**, *30*, 44–49.	Mismatch with inclusion criteria
39	Fagan, A.; Lea, J.; Parker, V. Conflict, confusion and inconsistencies: Pre-registration nursing students' perceptions and experiences of speaking up for patient safety. *Nurs. Inq*. **2020**, *28*, e12381.	Mismatch with inclusion criteria
40	Farley, D.; Zheng, H.; Rousi, E.; Leotsakos, A. Field Test of the World Health Organization Multi-Professional Patient Safety Curriculum Guide. *PloS. One.* **2015**, *10*, e0138510.	Mismatch with inclusion criteria
41	Ferguson, C.C.; Lamb, G. A Scholarly Pathway in Quality Improvement and Patient Safety. *Acad. Med.* **2015**, *90*, 1358–1362.	Mismatch with inclusion criteria
42	Ferraro, K.; Zernzach, R.; Maturo, S.; Nagy, C.; Barrett, R.. Chief of Residents for Quality Improvement and Patient Safety: A Recipe for a New Role in Graduate Medical Education. *Mil. Med*. **2017**, *182*, e1747–e1751.	Mismatch with inclusion criteria
43	Flin, R.; Patey, R.; Jackson, J.; Mearns, K.; Dissanayaka, U. Year 1 medical undergraduates' knowledge of and attitudes to medical error. *Med. Educ.* **2009**, *43*, 1147–1155.	Mismatch with inclusion criteria (validation study)
44	Gantt, L.T.; Webb-Corbett, R. Using simulation to teach patient safety behaviors in undergraduate nursing education *J. Nurs. Educ*. **2010**, *49*, 48–51.	Full text not available
45	Gaupp, R.; Dinius, J.; Drazic, I.; Körner, M. Long-term effects of an e-learning course on patient safety: A controlled longitudinal study with medical students. *PloS. One.* **2019**,*14*, doi:10.1371/journal.pone.0210947.	Mismatch with inclusion criteria
46	Gaupp, R.; Fabry, G.; Körner, M. Self-regulated learning and critical reflection in an e-learning on patient safety for third-year medical students- *Int. J. Med. Educ*. **2018**, *9*, 189–194.	Mismatch with inclusion criteria
47	Gaupp, R.; Körner, M.; Fabry, G. Effects of a case-based interactive e-learning course on knowledge and attitudes about patient safety: a quasi-experimental study with third-year medical students. *BMC. Med. Educ.* **2016**, *16*, 172.	Mismatch with inclusion criteria
48	Genuardi, F.J.; Kelly, M.N.; Rosenberg, E.I.; Black, E.W.; Dawson, K. Teaching Students How to Improve Safety and Quality in Two Children's Hospitals: Building a Pediatric Clerkship Patient Safety and Quality Experience. *Acad. Pediatr.* **2019**, *19*, 712–715.	Mismatch with inclusion criteria
49	Gill, A.C.; Cowart, J.B.; Hatfield, C.L.; Dello Stritto, R.A.; Landrum, P.; Ismail, N.; Nelson, E.A.; Teal, C.R. Patient Safety Interprofessional Training for Medical, Nursing, and Pharmacy Students. *MedEdPORTAL*. **2017**, *13*, 10595.	Mismatch with inclusion criteria
50	Ginsburg, L.; Castel, E.; Tregunno, D.; Norton, P.G. The H-PEPSS: an instrument to measure health professionals' perceptions of patient safety competence at entry into practice. *BMJ. Qual. Saf.* **2012**, *21*, 676-684.	Mismatch with inclusion criteria (validation study)
51	Ginsburg, L.R.; Tregunno, D.; Norton, P.G. Self-reported patient safety competence among new graduates in medicine, nursing and pharmacy. *BMJ. Qual. Saf.* **2013**, *22*, 147–154.	Mismatch with inclusion criteria
52	Ginsburg, L.R.; Tregunno, D.; Norton, P.G.; Smee, S.; de Vries, I.; Sebok, S.S.; VanDenKerkhof, E.G.; Luctkar-Flude, M.; Medves, J. Development and testing of an objective structured clinical exam (OSCE) to assess socio-cultural dimensions of patient safety competency. *BMJ. Qual. Saf.* **2015**, *24*, 188–194.	Mismatch with inclusion criteria
53	Gleason, K.T.; VanGraafeiland, B.; Commodore-Mensah, Y.; Walrath, J.; Immelt, S.; Ray, E.; Dennison Himmelfarb C.R. The impact of an innovative curriculum to introduce patient safety and quality improvement content. *BMC. Med. Educ*. **2019**, *19*, 156.	Mismatch with inclusion criteria
54	Goolsarran, N.; Hamo, C.E.; Lane, S.; Frawley, S.; Lu, W.H. Effectiveness of an interprofessional patient safety team-based learning simulation experience on healthcare professional trainees. *BMC. Med. Educ*. **2018**, *18*, 192.	Mismatch with inclusion criteria
55	Gopee, S. Enhancing patient safety: empowering students to speak up. *Br. J. Nurs*. **2020**, *29*, 712.	Full-text not available
56	Gantt, L.T.; Webb-Corbett, R. Using simulation to teach patient safety behaviors in undergraduate nursing education. *J. Nurs. Educ.* **2010**, *49*, 48–51.	Full text not available
57	Halbach, J.L.; Sullivan, L.L. Teaching medical students about medical errors and	Mismatch with

	patient safety: evaluation of a required curriculum. *Acad. Med.* **2005**, *80*, 600-606.	inclusion criteria
58	Hall, L.W.; Scott, S.D.; Cox, K.R.; Gosbee, J.W.; Boshard, B.J.; Moylan, K.; Dellsperger, K.C. Effectiveness of patient safety training in equipping medical students to recognise safety hazards and propose robust interventions. *Qual. Saf. Health. Care.* **2010,***19*, 3–8.	Mismatch with inclusion criteria
59	Harendza, S.; Soll, H.; Prediger, S.; Kadmon, M.; Berberat, P.O.; Oubaid, V. Assessing core competences of medical students with a test for flight school applicants. *BMC. Med. Educ.* **2019**,*19*, 9.	Mismatch with inclusion criteria
60	Hayes, A.J.; Roberts, P.; Figgins, A.; Pool, R.; Reilly, S.; Roughley, C.; Salter, T.; Scott, J.; Watson, S.; Woodside, R.; Patel, V. Improving awareness of patient safety in a peer-led pilot educational programme for undergraduate medical students. *Educ. Health. (Abingdon).* **2014**, *27*, 213–216.	Mismatch with inclusion criteria
61	Hindle, D.; Haraga, S.; Radu, C.P.; Yazbeck, A.M. What do health professionals think about patient safety? *J. Public. Health.* **2008**,*16*, 87–96.	Mixed population
62	Huang, F.F.; Shen, X.Y.; Chen, X.L.; He, L.P.; Huang, S.F.; Li, J.X. Self-reported confidence in patient safety competencies among Chinese nursing students: a multi-site cross-sectional survey. *BMC. Med. Educ.* **2020**, *20*, 32.	Mismatch with inclusion criteria
63	Hull, L.; Arora, S.; Amaya, A.C.; Wheelock, A.; Gaitán-Duarte, H.; Vincent, C.; Sevdalis, N. Building global capacity for patient safety: a training program for surgical safety research in developing and transitional countries. *Int. J. Surg.* **2012**,*10*, 493–499.	Mixed population
64	Hwang, J.I.; Yoon, T.Y.; Jin, H.J.; Park, Y.; Park, J.Y.; Lee, B.J. Patient safety competence for final-year health professional students: Perceptions of effectiveness of an interprofessional education course. *J. Interprof. Care.* **2016**, *30*, 732–738.	Mismatch with inclusion criteria
65	Hwang, J.I. What are hospital nurses' strengths and weaknesses in patient safety competence? Findings from three Korean hospitals. *Int. J. Qual. Health. Care.* **2015**, *27*, 232–238.	Mismatch with inclusion criteria
66	Ironside, P.M.; Jeffries, P.R.; Martin, A. Fostering patient safety competencies using multiple-patient simulation experiences. *Nurse. Outlook.* **2009**, *57*, 332–337.	Mismatch with inclusion criteria
67	Jansson, P.S.; An-Grogan, Y.; Eller, S.G.; Woods, D.M.; Kontrick, A.V.; Salzman, D.H. A needs assessment in patient safety education for fourth-year medical students. *Am. J. Med. Qual.* **2015**, *30*, 601.	Mismatch with inclusion criteria
68	Jenkins, S.; Blake, J.; Brandy-Webb, P.; Ashe, W. Teaching patient safety in simulated learning experiences. *Nurse. Educ.* **2011**, *36*, 112–117.	Mismatch with inclusion criteria
69	Jha, V.; Buckley, H.; Gabe, R.; Kanaan, M.; Lawton, R.; Melville, C.; Quinton, N.; Symons, J.; Thompson, Z.; Watt, I.; Wright, J. Patients as teachers: a randomised controlled trial on the use of personal stories of harm to raise awareness of patient safety for doctors in training. *BMJ. Qual.Saf.* **2015**, *24*, 21–30.	Mismatch with inclusion criteria
70	Jin, J.; Yi Y.J. Patient safety competency and the new nursing care delivery model. *J. Nurs. Manag.* **2019**, *27*, 1167–1175.	Mismatch with inclusion criteria
71	Kahriman, I.; Ozturk, H.; Bahcecik, N.; Sokmen, S.; Kucuk, S.; Calbayram, N.; Altundag, S. The effect of theoretical and simulation training on medical errors of nurse students in Karadeniz Technical University, Turkey. *JPMA.* **2018,** *68*, 1636–1643.	Mismatch with inclusion criteria
72	Kerfoot, B.C.; Conlin, P.R.; Travison, T.; McMahon, G.T. Patient safety knowledge and its determinants in medical trainees. *J. Gen. Intern. Med.* **2007**, *22*, 1150–1154.	Mismatch with inclusion criteria
73	Kiesewetter, J.; Kager, M.; Fischer, M.R.; Kiesewetter, I. Validation of a German short version of the Attitudes towards Patient Safety Questionnaire (G-APSQshort) for the measurement of undergraduate medical students' attitudes to and needs for patient safety. *GMS. J. Med. Educ.* **2017**, *34*, Doc8, doi:10.3205/zma001085.	Mismatch with inclusion criteria (validation study)
74	Killam, L.A.; Montgomery, P.; Raymond, J.M.; Mossey, S.; Timmermans, K.E.; Binette, J. Unsafe clinical practices as perceived by final year baccalaureate nursing students: Q methodology. *BMC. Nursing.* **2012**, *11*, 26.	Mismatch with inclusion criteria
75	Kim, Y.M.; Yoon, Y.S.; Hong, H.C.; Min, A. Effects of a patient safety course using a flipped classroom approach among undergraduate nursing students: A quasi-experimental study. *Nurse. Educ. Today.* **2019**, *79*, 180–187.	Mismatch with inclusion criteria
76	Know, A.W.C.; Ang, B.L.S.; Chong, C.S.; Tan, W.B.; Menon, K.R. Innovative Patient Safety Curriculum Using iPAD Game (PASSED) Improved Patient Safety Concepts in Undergraduate Medical Students. *World. J. Surg.* **2016**, *40*, 2571–2580.	Mismatch with inclusion criteria
77	Kutaimy, R.; Zhang, L.; Blok, D.; Kelly, R.; Kovacevic, N.; Levoska, M.; Gadivemula, R.;	Mismatch with

	Levine, D. Integrating patient safety education into early medical education utilizing cadaver, sponges, and an inter-professional team. *BMC. Med. Educ.* **2018**,*18*, 215.	inclusion criteria
78	Langlois, S. Developing an appreciation of patient safety: analysis of interprofessional student experiences with health mentors. *Perspect. Med. Educ.* **2016**, *5*, 88–94.	Mismatch with inclusion criteria
79	Larramendy-Magnin, S.; Anthoine, E.; L'Heude, B.; Leclère, B.; Moret, L. Refining the medical student safety attitudes and professionalism survey (MSSAPS): adaptation and assessment of patient safety perception of French medical residents. *BMC. Med. Educ*. **2019**, *19*, 222.	Mismatch with inclusion criteria (validation study)
80	Lee, B.O.; Liang, H.F.; Chu, T.P.; Hung, C.C. Effects of simulation-based learning on nursing student competences and clinical performance. *Nurse. Educ. Pract*. **2019**, *41*, 102646.	Mismatch with inclusion criteria
81	Lee, N.J.; Jang, H.; Park, S.Y. Patient safety education and baccalaureate nursing students' patient safety competency: A cross-sectional study. *Nurs. Health. Sci.* **2016**, *18*, 163–171.	Mismatch with inclusion criteria
82	Lee, S.E.; Lee, M.H.; Peters, A.B.; Gwon, S.H. Assessment of Patient Safety and Cultural Competencies among Senior Baccalaureate Nursing Students. *Int. J. Environ. Res. Public. Health.* **2020**,*17*, 4225.	Mismatch with inclusion criteria
83	Leung, G.K.K.; Ang, S.B.L.; Lau, T.C.; Neo, H.J.; Patil, N.G.; Ti, L.K. Patient safety culture among medical students in Singapore and Hong Kong. *Singapore. Med. J*. **2013**, *54,* 501–505.	Mismatch with inclusion criteria
84	Leung, G.K.K.; Patil, N.G.; Ip, M.S.M. Introducing patient safety to undergraduate medical students-a pilot program delivered by health care administrators. *Med. Teach.* **2010,** *32*, e547–551.	Mismatch with inclusion criteria
85	Li, G.; Tao, H.B.; Liao, J.Z.; Tang, J.H.; Peng, F.; Shu, Q.; Li, W.G.; Tu, S.G.; Chen, Z. Patient safety education among chinese medical undergraduates: An empirical study. *J. Huazhong. Univ. Sci. Technolog. Med. Sci*. **2016**, *36*, 780–784.	Mismatch with inclusion criteria
86	Li, L.; Duan, Y.; Chen, P.; Li, J.; Mao, X.; Barraclough, B.H.; Zhang, M. Knowledge, skills, and attitudes of medical students to patient safety: a cross-sectional pilot investigation in China. *J. Evid. Based. Med*. **2012**, *5*, 124–133.	Mismatch with inclusion criteria (validation study)
87	Liao, J.M.; Etchegaray, J.M.; Williams, S.T.; Berger, D.H.; Bell, S.K.; Thomas, E.J. Assessing medical students' perceptions of patient safety: the medical student safety attitudes and professionalism survey. *Acad. Med*. **2014**, *89*, 343–351.	Mismatch with inclusion criteria (testing of psychometric properties of questionnaire)
88	Lira, V.L.; Campelo, S.M.A.; Branco, N.F.L.C.; Carvalho, H.E.F.; Andrade, D.; Ferreira, A.M.; Ribeiro, I.P. Patient safety climate from the nursing perspective. *Rev. Bras. Enferm*. **2020**, *73*, e20190606.	Mismatch with inclusion criteria
89	Lukewich, J.; Edge, D.S.; Tranmer, J.; Raymond, J.; Miron, J.; Ginsburg, L.; VanDenKerkhof, E. Undergraduate baccalaureate nursing students' self-reported confidence in learning about patient safety in the classroom and clinical settings: an annual cross-sectional study (2010-2013). *Int. J. Nurs. Stud*. **2015**, *52*, 930–938.	Mismatch with inclusion criteria
90	Lochner, L.; Girardi, S.; Pavcovich, A.; Meier, H.; Mantovan, F.; Ausserhofer, D. Applying interprofessional Team-Based Learning in patient safety: a pilot evaluation study. *BMC. Med. Educ.* **2018**, *18*, 48.	Mismatch with inclusion criteria
91	Lypson, M.L.; Gosbee, J.W.; Andreatta, P. Assessing the patient safety knowledge and experience of trainees. *Med. Educ.* **2008**, *42*, 1133–1134.	Mismatch with inclusion criteria
92	Maatman, T.C.; Prigmore, H.; Williams, J.S.; Fletcher, K.E. Patient safety superheroes in training: using a comic book to teach patient safety to residents. *BMJ. Qual. Saf.* **2019,** *28*, 934–938.	Mismatch with inclusion criteria
93	Madigosky, W.S.; Headrick, L.A.; Nelson, K.; Cox, K.R.; Anderson, T. Changing and sustaining medical students' knowledge, skills, and attitudes about patient safety and medical fallibility. *Acad. Med.* **2006**, *81*, 94–101.	Mismatch with inclusion criteria
94	Mansour, M. Factor analysis of nursing students' perception of patient safety education. *Nurse. Educ. Today.* **2015**, *35*, 32–37.	Mismatch with inclusion criteria (validation study)
95	Mansour, M.; Skull, A.; Parker, M. Evaluation of World Health Organization Multi-Professional Patient Safety Curriculum Topics in Nursing Education: Pre-test, post-test,	Mismatch with inclusion criteria

	none-experimental study. *J. Prof. Nurs.* **2015**, *31*, 432–439.	
96	Maxwell, K.L.; Wright, V.H. Evaluating the Effectiveness of Two Teaching Strategies to Improve Nursing Students' Knowledge, Skills, and Attitudes About Quality Improvement and Patient Safety. *Nurs. Educ. Perspect*. **2016**, *37*, 291–292.	Mismatch with inclusion criteria
97	Mbuthia, N.N.; Moleki, M.M. Preregistration nursing students' perceived confidence in learning about patient safety in selected Kenyan universities. *Curationis*. **2019**, *42*, e1–e7.	Mismatch with inclusion criteria
98	McCarthy, S.E.; O'Boyle, C.A.; O'Shaughnessy, A.; Walsh, G. Online patient safety education programme for junior doctors: is it worthwhile? *Ir. J. Med. Sci.* **2016**, *185*, 51–58.	Mismatch with inclusion criteria
99	McKee, R.; Sussman, A.L.; Nelson, M.T.; Russell, J.C. Using Qualitative and Quantitative Assessment to Develop a Patient Safety Curriculum for Surgical Residents. *J. Surg. Educ.* **2016**, *73*, 529–535.	Mismatch with inclusion criteria
100	McPherson, S.; Wendler, M.C. Safe clinical practice in pre-licensure nursing students: A concept analysis. *Nurs. Forum*. **2020**, *55*, 513–522.	Mismatch with inclusion criteria
101	Miller, M.E.; Patel, A.; Schindler, N.; Hirsch, K.; Ming, M.; Weber, S.; Turner, P.; Howell, M.D.; Arora, V.M.; Oyler, J.L. Bridging the Gap: Interdepartmental Quality Improvement and Patient Safety Curriculum Created by Hospital Leaders, Faculty, and Trainees. *J. Grad. Med. Educ.* **2018**, *10*, 566–572.	Mismatch with inclusion criteria
102	Moazez, M.; Miri, S.; Foroughameri, G.; Farokhzadian J. Nurses' perceptions of systems thinking and safe nursing care: A cross-sectional study. *J. Nurs. Manag*. **2020**, *28*, 822–830.	Mismatch with inclusion criteria
103	Montgomery, P.; Killam, L.; Mossey, S.; Heerschap, C. Third year nursing students' viewpoints about circumstances which threaten safety in the clinical setting. *Nurs. Educ. Today.* **2014**, *34*, 271–276.	Mismatch with inclusion criteria
104	Moskowitz, E.; Veloski, J.J.; Fields, S.K.; Nash, D.B. Development and evaluation of a 1-day interclerkship program for medical students on medical errors and patient safety. *Am. J. Med. Qual.* **2007**, *22*, 13–17.	Mismatch with inclusion criteria
105	Mookherjee, S.; Ranji, S.; Neeman, N.; Sehgal, N. An advanced quality improvement and patient safety elective. *Clin. Teach.* **2013**,*10*, 368–373.	Mismatch with inclusion criteria
106	Murray, M.; Sundin, D.; Cope, V. New graduate nurses' understanding and attitudes about patient safety upon transition to practice. *J. Clin. Nurs.* **2019**, *28*, 2543–2552.	Mismatch with inclusion criteria
107	Myung, S.J.; Shin, J.S.; Kim, J.H.; Roh, H.; Kim, Y.; Kim, J.; Lee, S.I.; Lee, J.H.; Kim, S.W. The patient safety curriculum for undergraduate medical students as a first step toward improving patient safety. *J. Surg. Educ*. **2012**, *69*, 659–664.	Mismatch with inclusion criteria
108	Noviyanti, L.W.; Handiyani, H.; Gayatri, D. Improving the implementation of patient safety by nursing students using nursing instructors trained in the use of quality circles. *BMC. Nurs.* **2018,** *17*, 53.	Mismatch with inclusion criteria
109	Oates, K.; Wilson, I.; Hu, W.; Walker, B.; Nagle, A.; Wiley, J. Changing medical student attitudes to patient safety: a multicentre study. *BMC. Med. Educ*. **2018**,*18*, 205.	Mismatch with inclusion criteria
110	O'Keeffe, V.; Boyd, C.; Phillips, C.; Oppert, M. Creating safety in care: Student nurses' perspectives. *Appl. Ergon*. **2020**, *90*, 103248.	Mismatch with inclusion criteria
111	Ortiz de Elguea, J.; Orkaizagirre-Gómara, A.: Sánchez De Miguel, M.; Urcola-Pardo, F.; Germán-Bes, C.; Lizaso-Elgarresta, I. Adapting and validating the Hospital Survey on Patient Safety Culture (HSOPS) for nursing students (HSOPS-NS): A new measure of Patient Safety Climate. *Nurs. Educ. Today.* **2019**, *75*, 95–103.	Mismatch with inclusion criteria (validation study)
112	Palmer, J.C.; Blanchard, J.R.; Jones, J.; Bailey, E. Attitudes of dental undergraduate students towards patient safety in a UK dental school. *Eur. J. Dent. Educ.* **2019,** *23*, 127–134.	Mismatch with inclusion criteria
113	Parente, V.; Feeney, C.; Page, L.; Johnson, S.; Porada, K.; Cheifetz, I.; Stephany, A. Sustained Impact of a Pediatric Resident-Led Patient Safety Council. *J. Patient. Saf*. **2018**, doi:10.1097/PTS.0000000000000495.	Full-text not available
114	Patey, R.; Flin, R.; Cuthbertson, B.H.; MacDonald, L.; Mearns, K.; Cleland, J.; Williams, D. Patient safety: helping medical students understand error in healthcare. *Qual. Saf. Health. Care.* **2007**,*16*, 256-259.	Mismatch with inclusion criteria
115	Pearson, P.; Steven, A.; Howe, A.; Sheikh, A.; Ashcroft, D.; Smith, P.; Patient Safety Education Study Group. Learning about patient safety: organizational context and culture in the education of health care professionals. *J. Health. Serv. Res. Policy.*	Mismatch with inclusion criteria

	**2010**,*15*, 4–10.	
116	Peterson-Graziose, V.; Bryer, J. Assessing Student Perceptions of Quality and Safety Education for Nurses Competencies in a Baccalaureate Curriculum. *J. Nurs. Educ*. **2017**, *56*, 435–438.	Mismatch with inclusion criteria
117	Raymond-Dufresne, E.; Brazil, V.; Johnson, P.L.; Nielson, T.L. Pre-clinical medical students' perceptions of their patient safety skills in a simulated emergency department. *Emerg. Med. Australas.* **2016**, *28*, 325–328.	Mismatch with inclusion criteria
118	Raymond, J.; Medves, J.; Godfrey, C. Perspectives on Patient Safety Among Practical Nursing Students. *Can. J. Nurs. Res*. **2016**, *48*, 41–47.	Mismatch with inclusion criteria
119	Reznek, M.A.; Digiovine, B.; Kromrei, H.; Levine, D.; Wiese-Rometsch, W.; Schreiber, M. Quality Education and Safe Systems Training (QuESST): Development and Assessment of a Comprehensive Cross-Disciplinary Resident Quality and Patient Safety Curriculum. *J. Grad. Med. Educ.* **2010**, *2*, 222–227.	Mismatch with inclusion criteria
120	Rinke, M.L.; Mock, C.K.; Persing, N.M.; Sawyer, M.; Haut, E.R.; Neufeld, N.J.; Nagy, P. The Armstrong Institute Resident/Fellow Scholars: A Multispecialty Curriculum to Train Future Leaders in Patient Safety and Quality Improvement. *Am. J. Med. Qual*. **2016**, *31*, 224–232.	Mismatch with inclusion criteria
121	Robson, J.; de Wet, C.; McKay, J.; Bowie, P. Do we know what foundation year doctors think about patient safety incident reporting? Development of a web based tool to assess attitude and knowledge. *Postgrad. Med. J.* **2011**, *87*, 750–756.	Mismatch with inclusion criteria
122	Roh, H.; Park, S.J.; Kim, T. Patient safety education to change medical students' attitudes and sense of responsibility. *Med. Teach*. **2015,** *37*, 908–914.	Mismatch with inclusion criteria
123	Rosentreter, M.; Groß, D.; Schäfer, G. Pilot project "Patient-Safety" in Medical Education. *GMS. Z. Med. Ausbild*. **2011**, *28*, Doc12, doi:10.3205/zma000724.	Mismatch with inclusion criteria
124	Ross, J.G.; Bruderle, E. Effects of Active, Student-Centered Teaching Strategies on Nursing Students' Knowledge, Skills, Attitudes, and Comfort Related to Patient Safety. *Nurse. Educ.* **2018**, *43*, 2–3.	Mismatch with inclusion criteria
125	Ryan, E.L.J.; Jackson, D.; Woods, C.; Usher, K.J. Pre-registration nursing students' perceptions and experience of intentional rounding: A cross-sectional study. *Nurse. Educ. Pract.* **2020**, *42*, 102691.	Mismatch with inclusion criteria
126	Schnall, R.; Stone, P.; Currie, L.; Desjardins, K.; John, R.M.; Bakken, S. Development of a self-report instrument to measure patient safety attitudes, skills, and knowledge. *J. Nurs. Scholarsh.* **2008**, *40,* 391–394.	Mismatch with inclusion criteria (validation study)
127	Shah, K.P.; Goyal, S.; Ramachandran, V.; Kohn, J.R.; Go, J.A.; Wiley, Z.; Moturu, A.; Namireddy, M.K.; Kumar, A.; Jacobs, R.C. et al. Efficacy of quality improvement and patient safety workshops for students: a pilot study. *BMC. Med. Educ.* **2020**, *20*, 126.	Mismatch with inclusion criteria
128	Shaw, T.J.; Pernar, L.I.; Peyre, S.E.; Helfrick, J.F.; Vogelgesang, K.R.; Graydon-Baker, E.; Chretien, Y., Brown, E.J., Nicholson, J.C.; Heit, J.J.; Co, J.P.T., Gandhi, T. Impact of online education on intern behaviour around joint commission national patient safety goals: a randomised trial. *BMJ. Qual. Saf.* **2012,** *21*, 819–825.	Mismatch with inclusion criteria
129	Silkens, M.E.W.M.; Arah, O.A.; Wagner, C.; Scherpbier, A.J.J.A.; Heineman, M.J.; Lombarts, K.M.J.M.H. The Relationship Between the Learning and Patient Safety Climates of Clinical Departments and Residents' Patient Safety Behaviors. *Acad. Med*. **2018**, *93*,1374–1380.	Mismatch with inclusion criteria
130	Sorokin, R.; Riggio, J,M.; Moleski, S.; Sullivan, J. Physicians-in-training attitudes on patient safety: 2003 to 2008. *J. Patient. Saf.* **2011**, *7*, 133–138.	Mismatch with inclusion criteria (follow-up data)
131	Stevanin, S.; Bressan, V.; Bulfone, G.; Zanini, A.; Dante, A.; Palese, A. Knowledge and competence with patient safety as perceived by nursing students: The findings of a cross-sectional study. *Nurs. Educ. Today.* **2015**, *35*, 926–934.	Mismatch with inclusion criteria
132	Stomski, N.; Gluyas, H.; Andrus, P.; Williams, A.; Hopkins, M.; Walters, J.; Sandy, M.; Morrison, P. The influence of situation awareness training on nurses' confidence about patient safety skills: A prospective cohort study. *Nurs. Educ. Today.* **2018**, *63*, 24–28.	Mismatch with inclusion criteria
133	Stueven, J.; Sklar, D.P.; Kaloostian, P.; Jaco, C.; Kalishman, S.; Wayne, S.; Doering, A.; Gonzales, D. A resident-led institutional patient safety and quality improvement process. *Am. J. Med. Qual.* **2012,** *27*, 369–376.	Mismatch with inclusion criteria
134	Sukkari, S.R.; Sasich, L.D.; Tuttle, D.A.; Abu-Baker, A.M.; Howell, H. Development and	Mismatch with

	evaluation of a required patient safety course. *Am. J. Pharm. Educ*. **2008**, *72*, 65.	inclusion criteria
135	Sullivan, D.T.; Hirst, D.; Cronenwett, L. Assessing quality and safety competencies of graduating prelicensure nursing students. *Nurs. Outlook.* **2009**, *57*, 323–331.	Mismatch with inclusion criteria
136	Swamy, L.; Badke, C.; Suguness, A.; McKeegan, J.; Kirkham, K.; Borges, N.J. Student Perceptions of Clinical Quality and Safety. *Am. J. Med. Qual.* **2016**, *31*, 601.	Mismatch with inclusion criteria
137	Taskiran, G.; Eskin Bacaksiz, F.; Harmanci Seren, A.K. Psychometric testing of the Turkish version of the Health Professional Education in Patient Safety Survey: H-PEPSSTR. *Nurse. Educ. Pract. 2019*, *42*,102640.	Mismatch with inclusion criteria (validation study)
138	Tella, S.; Smith, N.J.; Partanen, P.; Jamookeeah, D.; Lamidi, M.L.,; Turunen, H. Learning to ensure patient safety in clinical settings: comparing Finnish and British nursing students' perceptions. *J. Clin. Nurs.* **2015,** *24*, 2954–2964.	Mismatch with inclusion criteria
139	Tella, S.; Smith, N.J.; Partanen, P.; Turunen, H. Learning Patient Safety in Academic Settings: A Comparative Study of Finnish and British Nursing Students' Perceptions. *Worldviews. Evid. Based. Nurs*. **2015**, *12*, 154–164.	Mismatch with inclusion criteria
140	Thom, K.A.; Heil, E.L.; Croft, L.D.; Duffy, A.; Morgan, D.J.; Johantgen, M. Advancing interprofessional patient safety education for medical, nursing, and pharmacy learners during clinical rotations. J. *Interprof. Care*. **2016**, *30*, 819–822.	Mismatch with inclusion criteria
141	Trinkley, K.E.; Van Matre, E.T.; Mueller, S.W.; Page, R.L.; Nair, K. Perceived Benefit of Teaching Patient Safety to Pharmacy Students by Integrating Classroom Teaching With Introductory (IPPE) Visits. *J. Pharm. Pract.* **2017**, *30*, 115–120.	Mismatch with inclusion criteria
142	Usher, K.; Woods, C.; Parmenter, G.; Hutchinson, M.; Mannix, J.; Power, T.; Chaboyer, W.; Latimer, S.; Mills, J.; Siegloff, L.; Jackson, D. Self-reported confidence in patient safety knowledge among Australian undergraduate nursing students: A multi-site cross-sectional survey study. *Int. J. Nurs. Stud.* **2017**, *71*, 89–96.	Mismatch with inclusion criteria
143	Vaismoradi, M.; Bondas, T.; Jasper, M.; Turunen, H. Nursing students' perspectives and suggestions on patient safety--implications for developing the nursing education curriculum in Iran. *Nurs. Educ. Today.* **2014**, *34*, 265–270.	Mismatch with inclusion criteria
144	Vaismoradi, M.; Salsali, M.; Marck, P. Patient safety: nursing students' perspectives and the role of nursing education to provide safe care. *Int. Nurs. Rev.* **2011**, *58*, 434–442.	Mismatch with inclusion criteria
145	VanDenKerkhof, E.; Sears, N.; Edge,D.S.; Tregunno, D.; Ginsburg, L. Patient safety in practical nurses' education: A cross-sectional survey of newly registered practical nurses in Canada. *Nurs. Educ. Today.* **2017**, *51*, 48–56.	Mismatch with inclusion criteria
146	Vyas, D.; McCulloh, R.; Dyer, C.; Gregory, G.; Higbee, D. An interprofessional course using human patient simulation to teach patient safety and teamwork skills. *Am. J. Pharm. Educ*. **2012**, *76*, 71.	Mismatch with inclusion criteria
147	Vohra, P.D.; Johnson, J.K.; Daugherty, C.K.; Wen, M.; Barach, P. Housestaff and medical student attitudes toward medical errors and adverse events. *Jt. Comm. J. Qual. Patient. Saf*. **2007**, *33*, 493–501.	Mismatch with inclusion criteria
148	Wagner, D.P.; Hoppe, R.B.; Lee, CP. The patient safety OSCE for PGY-1 residents: a centralized response to the challenge of culture change. *Teach. Learn. Med*. **2009**, *21*, 8–14.	Mismatch with inclusion criteria
149	Walker, D.; Altmiller, G.; Hromadik, L.; Barkell, N.; Barker, N.; Boyd, T.; Compton, M.; Cook, P.; Curia, M.; Hays, D. et al. Nursing Students' Perceptions of Just Culture in Nursing Programs: A Multisite Study. *Nurse. Educ.* **2020**, *45*, 133–138.	Mismatch with inclusion criteria
150	Wallin, C.J.; Kalman, S.; Sandelin, A.; Färnert, M.L.; Dahlstrand, U.; Jylli, L. Creating an environment for patient safety and teamwork training in the operating theatre: A quasi-experimental study. *Med. Teach.* **2015**, *37*, 267–276.	Wrong focus
151	Walpola, R.L.; Fois, R.A.; Carter, S.R.; McLachlan, A.J.; Chen, T.F. Validation of a survey tool to assess the patient safety attitudes of pharmacy students. *BMJ. Open.* **2015**, *5*, e008442.	Mismatch with inclusion criteria (validation study)
152	Walpola, R.L.; Fois, R.A.; McLachlan, A.J.; Chen, T.F. Evaluating the effectiveness of a peer-led education intervention to improve the patient safety attitudes of junior pharmacy students: a cross-sectional study using a latent growth curve modelling approach. *BMJ Open* **2015**, *5*, e010045.	Mismatch with inclusion criteria
153	Walpola, R.L.; Fois, R.A.; McLachlan, A.J.; Chen, T.F. Evaluating the Effectiveness of an Educational Intervention to Improve the Patient Safety Attitudes of Intern Pharmacists. *Am. J. Pharm. Educ.* **2017**, *81*, 5.	Mismatch with inclusion criteria
154	Wakefield, J.G.; McLaws, M.L.; Whitby, M.; Patton, L. Patient safety culture: factors that	Mismatch with

	influence clinician involvement in patient safety behaviours. *Qual. Saf. Health. Care*. **2010**, *19*, 585–591.	inclusion criteria
155	Ward, M.; Ní Shé, E.; De Brún, A.; Korpos, C.; Hamza, M.; Burke, E.; Duffy, A.; Egan, K.; Geary, U.; Holland, C. et al. The co-design, implementation and evaluation of a serious board game 'PlayDecide patient safety' to educate junior doctors about patient safety and the importance of reporting safety concerns. *BMC. Med. Educ*. **2019**, *19*, 232.	Mismatch with inclusion criteria
156	Watanabe, H.; Makino, T.; Tokita, Y.; Kishi, M.; Lee, B.; Matsui, H.; Shinozaki, H.; Kama, A. Changes in attitudes of undergraduate students learning interprofessional education in the absence of patient safety modules: evaluation with a modified T-TAQ instrument. *J. Interprof. Care*. **2019**, *33*, 689–696.	Wrong focus
157	Willman, A.; Bjuresäter, K.; Nilsson, J. Newly graduated nurses' clinical competencies and need for further training in acute care hospitals. *J. Clin. Nurs*. **2020**, *29*, 2209–2220.	Mismatch with inclusion criteria

Supplementary File 4. PRISMA Flow Diagram

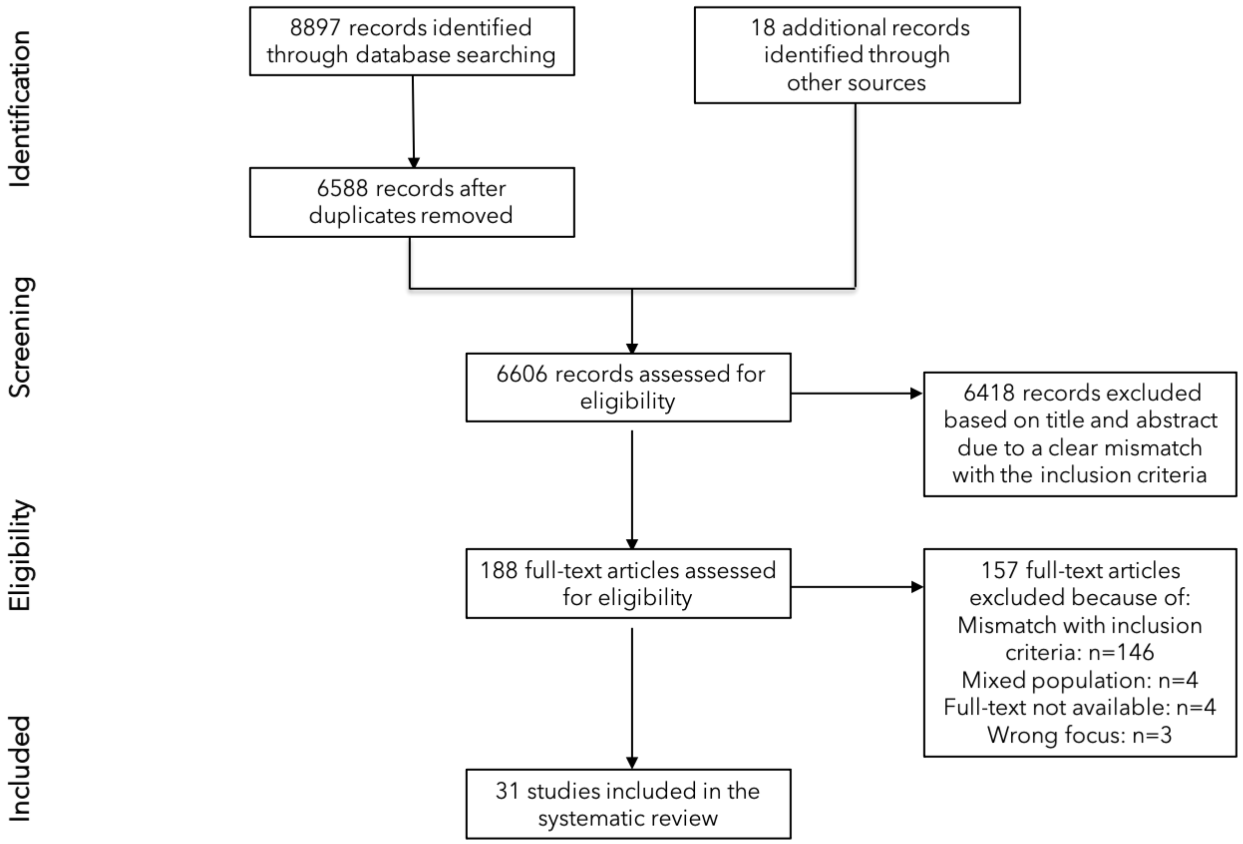

Supplementary File 5. Risk of bias assessment of included studies

MMAT Tool

Quantitative descriptive studies

	1. Is the sampling strategy relevant to address the research question?	2. Is the sample representative of the target population?	3. Are the measurements appropriate?	4. Is the risk of nonresponse bias low? (for case series and case report: are there complete data on the cases?)	5. Is the statistical analysis appropriate to answer the research question?
Almaramhy et al. (2011) [29]	✓	✗	✗	NA	✓
AlOlayan et al. (2020) [22]	✓	✗	✓	✓	✓
Alwhaibi et al. (2020) [13]	✓	✓	?	?	✓
Al-Surimi et al. (2018) [30]	✓	✓	?	✓	✓
Bari et al. (2017) [23]	✓	✓	?	✓	✗
Bowman et al. (2013) [31]	✓	✓	✗	✓	✓
Bump et al. (2015) [27]	✓	✗	✓	✓	✓
Bump et al. (2017) [28]	✓	✓	✓	✓	✓
Cauduro et al. (2017) [32]	✓	✓	✗	?	✓
De la Tassa et al. (2013) [33]	✓	✓	✗	✓	✓
Durani et al. (2013) [34]	✓	✓	✗	✗	✓
Escher et al. (2017) [35]	✓	✓	✓	✓	✓
Gropelli & Shanti (2018) [36]	✓	✗	✗	✓	✓
Kamran et al. (2018) [24]	✓	✓	?	✓	✓
Kamran et al. (2019) [25]	✓	✓	?	✓	✓
Kiesewetter et al. (2014) [37]	✓	✓	✓	NA	✓
Kong et al. (2019) [38]	✓	✓	✓	?	✓
Lee et al. (2018) [39]	✓	✓	✓	✓	✓
Leong et al. (2008) [40]	✓	✓	✓	✓	✗
Leung & Patil (2010) [41]	✓	✗	✗	✓	✗
Liu et al. (2018) [42]	✓	✓	?	✓	✗
Nabilou et al. (2015) [43]	✓	✓	✗	✓	✓
Nadarajan et al. (2020) [20]	✓	✓	?	✗	✓
Park et al. (2019) [44]	✓	✗	?	✓	✓
Parry et al. (2009) [45]	✓	✓	✓	✓	✓
Shah et al. (2015) [46]	✓	✗	✗	✓	✓
Sorokin et al. (2005) [47]	✓	✓	✗	✗	✓
Tegegn et al. (2017) [48]	✓	✓	?	✓	✓
Teigland et al. (2013) [49]	✓	✓	✗	✓	✗
Wetzel et al. (2012) [26]	✓	✓	✓	✓	✗
Yoshikawa et al. (2013) [50]	✓	✓	✗	✗	✗

✓= Yes; ✗= No; ? = Can't tell ; NA = not applicable

Supplementary File 6. Characteristics of included studies

Authors (year)	Country	Population	Sample size (N)	Outcome (instrument)	Main findings [%; n; mean±SD; *p*-values]	Differences across academic levels/years of study	Differences across specialties	Differences between genders
**Almaramhy et al., 2011 [29]**	Saudi Arabia	Undergraduate medical students	N=150	Knowledge level about patient safety issues and attitudes towards patient safety issues (ad hoc questionnaire)	'Agree' attitude:<br>▪ *Importance of patient safety topic*<br>o 'Patient safety is a global problem': 60.7%<br>o 'Most clinical errors are preventable': 50.0%<br>o 'Most health care staff make errors': 56.0%<br>o 'Most errors are out of staff control': 17.3%<br>o 'Competent physicians do not make errors': 82.0%<br>▪ *Problem solving*<br>o 'Patients play a role in preventing errors': 44.7%<br>o 'Support peers who make unintentional errors': 76.0%<br>o 'Not blaming peers for their own mistakes': 80.7%<br>o 'Cooperate with staff to resolve patient safety issues': 54.0%<br>o 'Willing to share information about clinical errors': 28.0%<br>o 'Do not hesitate to change practice habits to improve patient safety': 74.0%<br>▪ *Error Management*<br>o 'Willing to report errors whether or not patient was harmed': 52%<br>o 'No fear of negative consequences associated with reporting errors': 67.3%<br>o 'Likely to disclose an error to the faculty': 74.7%<br>o 'Likely to disclose error to the patient': 47.3%<br>▪ *Teaching patient safety issues*<br>o 'Teaching on patient safety in medical schools is necessary': 77.3%<br>o 'Patient safety should be a routine work of health care staff': 90.0%<br>o 'Continuous training of health care staff is necessary': 84.7%			
**AlOlayan et al., 2020 [22]**	Saudi Arabia	Dental students and interns	N=272	Awareness of patient safety culture and attitudes (SAQ)	'Agree' attitude:<br>• 'It is easy to ask questions when there is something that you do not understand' (*Teamwork climate*): 67.3%<br>• 'I have the needed support from other personnel for patient care': 66.5%<br>• 'I would feel safe being treated here as a patient': 62.5%<br>• 'Medical errors are handled appropriately in this clinical area' (*Safety climate*): 62.9%<br>• Most commonly agreed statement: 'I am proud to practice in this dental clinic' *(Job Satisfaction):* 75.4%<br>• Domains with the highest mean score: *Job satisfaction* (70.25±21.22) and *Work condition* (64.74±20.11)	• Significant increase in mean scores for *Teamwork climate* (p<0.001), *Safety* climate (p=0.002), *Perception of management* (p=0.002) and *Work condition* from the fourth year to the internship (p<0.001)<br>• Significant increase in mean SAQ score from junior classes to the internship (p<0.001)		• Significantly higher (p=0.004) mean score of *Stress recognition* in women than men<br>• No significant differences in the mean scores of SAQ between women and men
**Alwhaibi et al., 2020 [13]**	Saudi Arabia	Pharmacy students	N=347	Attitudes toward patient safety (APSQ-III)	'Agree' attitude:<br>• 'Training prepares respondents to understand the causes of medical errors': 55.3%<br>• 'Felt comfortable reporting errors they had made and talk openly to their supervisors': 56.2%<br>• 'Errors can be avoided by paying more attention at work: 34.2%<br>• 'All medical errors should be reported': 64.6%<br>• 'Teaching students about patient safety should be an important priority': 65.7%<br>• 'Learning about patient safety issues will enable them to become more effective': 64.3%			• Significantly more positive attitudes among women than men in the domains *Patient safety training received, Error reporting confidence, Working hours as error cause, Error inevitability, Team functioning, Patient involvement*

					'Disagree' attitude:<br>• 'A true professional does not make errors': 62%			*in reducing errors*<br>• Significantly more positive attitudes among men than women in the domain *Professional incompetence as error*<br>• No significant differences between women and men in the domain *Disclosure responsibility*
**Al-Surimi et al., 2018 [30]**	Saudi Arabia	Female dental students (students of dental sciences and students of dental hygiene)	N=211	Perception of patient safety culture (SAQ)	• Highest positive response in *Teamwork climate* for 'It is easy to ask questions if something is not understandable' (70.5%)<br>• Highest positive response in *Safety climate* for 'Received appropriate feedback about their performance' (60.6%)<br>• Highest positive response in *Job satisfaction* for 'I like my specialty' (75.5%)<br>• Highest positive response in *Stress recognition* for "I am less effective when I feel fatigued", 60.6%<br>• Highest positive response in *Perceived management support* for 'Clinical supervisor is doing a good job', 60.6%<br>• Highest positive response in *Working conditions* for 'I experience good collaboration with students in this clinical area', 62.4%<br>• Significantly more students had positive perceptions within *Job satisfaction* (65.6%) and *Working condition* (57%) than negative perceptions (p<0.001 and p=0.037, respectively)	• Significantly more positive perceptions in *Teamwork climate* in students in their third or fourth year of clinical training (58.1%) than students in their first or second year (35.9%, p=0.001)<br>• Students in their third or fourth years of clinical training 2.3 times more likely to have a positive perception of *Teamwork climate* than junior students (adjusted p=0.005)	• Dentistry students significantly more likely than dental students to have positive perceptions in each of the individual SAQ domains	
**Bari et al., 2017 [23]**	Pakistan	Postgraduate residents (and nurses)	N=150	Patient safety awareness (APSQ-IV)	• Highest scores for 'Patient safety is every one's responsibility' (mean score 6.16), 'Planning together to deal with a problem' (5.87) and 'Teaching students about patient safety' (5.85)<br>• Lowest score for 'I would feel comfortable reporting any errors other people had made, no matter how serious the outcome had been for the patient' (4.40)	• Significantly higher mean scores in positively worded questions in residents in the early years of residency than in residents in the last years of training (p=0.006)		
**Bowman et al., 2013 [31]**	United States of America	Fourth-year medical students	N=121	Student's perception of safety culture (ad hoc questionnaire)	'Agree' attitude:<br>• 'Clinical services were actively doing things to improve patient safety': 65%<br>• 'Attendings and residents addressed patient safety problems during the clerkships': 76% and 71%<br>• 'There was adequate clinical supervision of trainees': 70%<br>• 'Medical errors should be disclosed to patients': 80%<br>• 'Physicians are encouraged to disclose adverse events': 42%<br>• 'Would not speak up when witnessing an event that may negatively affect patient care': 56%<br>• 'Afraid to ask questions when things did not seem right': 55%<br>• 'Mistakes were held against respondents during the clerkship': 48%<br><br>• Highest rates for the domains *Teamwork, Supervisor Promoting Patient Safety,* and *Organizational Learning*<br>• Lowest rates for the domains *Communication openness, Non-punitive response to error,* and *Physician-to-physician handoffs*<br>• Team of providers and clinical service seen as having the greatest		• Average percent of positive responses for *Teamwork* was significantly higher for clinical experiences in internal medicine than in surgery (p<0.05)	

					influence on providing safe patient care (60% and 23%, respectively)<br>• Additional training in patient safety considered as beneficial for education<br>• Patient handoffs most prioritized learning need<br>• Coping with errors least prioritized learning need			
**Bump et al., 2015 [27]**	United States of America	Residents and fellows	N=955	Perceptions of patient safety culture (HSOPSC - The Agency for Healthcare Research and Quality Hospital Survey on Patient Safety Culture)	• Training programs and hospitals given an overall patient safety grade of 'very good'<br>• Higher scores in trainees than in national practitioners in *Supervisor/manager expectations and actions promoting patient safety* and *Staffing*<br>• Lower scores in trainees than in national practitioners in *Teamwork, Organizational learning-continuous learning, Management support for patient safety, Overall perceptions of patient safety, Feedback and communication about error*, and *Communication openness*	• No improvements of perceptions of patient safety culture with advancing postgraduate year		
**Bump et al., 2017 [28]**	United States of America	Residents and fellows (and general staff)	N=926	Perceptions of patient safety culture (HSOPSC - The Agency for Healthcare Research and Quality Hospital Survey on Patient Safety Culture)	• Significantly higher scores in trainees than general staff in four domains, including *Non-punitive response to error* (around 45% positive answers), *Staffing* (around 60%), *Supervisor/manager expectations and actions promoting patient safety* (around 80%), and *Teamwork within units* (around 60%) ($p<0.05$)<br>• Significantly lower scores in trainees than general staff in *Feedback and communication about error* (around 50%), *Frequency of event reporting* (around 45%), and *Overall perception of patient safety* (around 50%) ($p<0.05$)			
**Cauduro et al., 2017 [27]**	Brazil	Healthcare students	N=638	Understanding on patient safety (ad hoc questionnaire)	'Agree' attitude:<br>• 'There is a big difference between what the professionals know to be right and what is seen in practice': 81.3%<br>• 'When the error occurs, all of those involved should discuss the event': 90.1%<br>• 'For the human error analysis, it is important to know what the individual characteristics of the professional who made the mistake are': 69.7%<br>• 'After an error occurs, an effective prevention strategy is to work more carefully': 91.6%<br>• 'Workers should not tolerate working in places that do not offer suitable work conditions': 76.2%<br>• 'To implement preventive measures a systemic analysis of the facts should always be held': 86.4%<br>• 'Preventive measures need to be taken whenever anyone is injured': 80.2%<br><br>'Disagree' attitude:<br>• 'Making mistakes in health care is inevitable': 58.6%<br>• 'Competent professionals and committed students do not make mistakes': 60.8% and 61.6%, respectively<br>• 'Systems for reporting errors make little difference in reducing future errors': 77.4%<br><br>• No general consensus on 'I always perform internship activities in places that promote good practices for the promotion of patient safety' and 'Whenever I identify situations that need improvement, I receive support from the institution to implement measures to promote safe practices'			

**De la Tassa et al., 2013 [33]**	Spain	All healthcare trainees	N=359	Attitudes towards patient safety (Thomas Jefferson Survey on inpatient adverse events - adapted)	'Agree' attitude:<br>• 'Adverse events are preventable': 50%<br>• 'Training prepares respondents for prevention of adverse events: 40%<br>• 'Adverse events are a consequence of medical/nursing errors': 28.7% and 22.2%, respectively<br>• 'The level of patient responsibility for adverse events is high and relates to non-adherence to treatment': 54.4%<br>• 'Adverse events must be disclosed to patients/supervisors': 47.9% and 51.6%, respectively<br><br>• Trainees considered malpractice fear to interfere with the analysis of adverse events.	• Statistically significant differences by years of training only in questions related to improvements in techniques and procedures, with more advanced training leading to a significantly higher perception of importance (p<0.05)<br>• Significantly higher disagreement rate regarding involvement in groups for patient safety improvement in health care trainees in the initial years of training (p<0.05)		
**Durani et al., 2013 [34]**	United Kingdom	Foundation trainees, general practice trainees and hospital core and specialty trainees	N=527	Patient safety attitudes (Junior Doctor Patient Safety Attitudes and Safety Climate Questionnaire)	Attitudes:<br>• Highest proportion of 'correct' responses: 'Even the most experienced and competent doctors make errors' (99%'agree'/'correct', p<0.001)<br>• Lowest proportion of 'correct' responses: 'It is only important to disclose errors to patients if they have resulted in harm' (58% 'disagree'/'correct' response, p<0.001)<br>• Neutral attitude about 'If a junior doctor makes an error, the consultant should take most of the responsibility', 'I believe that filling in error reporting forms will help to improve patient safety'<br><br>Safety climate:<br>• Statements 'The senior managers in my hospital listen to me and care about my patient safety concerns' and 'Nurses on my ward are likely to criticize me for making mistakes' recorded a neutral attitude.<br>• The statement with the highest proportion of 'correct' response was 'Senior doctors in the department listen to me and care about my patient safety concerns' (70% agree, p<0.001).<br>• The statement with the lowest proportion endorsing the 'correct' response was 'Management is more interested in meeting performance targets than focusing on patient safety issues' (20% 'disagree'/'correct', p<0.001)	• Significantly greater proportion of junior trainees than seniors correctly responding to 'Medical error is a sign of incompetence' (p<0.001), and 'It is only important to disclose errors to patients if they have resulted in harm' (p=0.008)<br>• Significantly greater proportion of senior trainees correctly responding to 'Management is more interested in meeting performance targets than focusing on patient safety issues' (p<0.001), 'My suggestions about patient safety would be acted upon if I expressed them to management' (p<0.001), 'I know the proper channels to direct questions regarding patient safety' (p<0.001), 'The senior managers in my hospital listen to me and care about my patient safety concerns' (p<0.001), 'The senior doctors in my department listen to me and care about my	• Significantly lower number of surgical trainees than medical trainees correctly responding to 'The number of hours doctors work increases the likelihood of making errors' (p=0.035)<br>• Significantly lower number of surgical trainees than medical trainees 'correctly' responding to 'Medical error is a sign of incompetence' (p<0.001), 'Learning about patient safety is not as important as learning other more skill-based aspects of being a doctor' (p<0.001), and 'It is only important to disclose errors to patients if they have resulted in harm' (p<0.001)<br>• No statistically significant differences in responses between the	

						patient safety concerns' (p=0.002), and 'I would feel safe here being treated as an inpatient' (p=0.004)	medical and surgical trainee groups for perception of workplace safety climate	
**Escher et al., 2017 [35]**	Sweden	Medical students	N=56	Attitudes towards patient safety (APSQ-III)	Attitudes to patient safety total scores (mean, SD):<br>• *Patient safety training received*: 15.3 (2.5)<br>• *Error reporting confidence*: 14.2 (3.0)<br>• *Working hours as error cause*: 17.7 (2.8)<br>• *Error inevitability*: 18.0 (1.8)<br>• *Professional incompetence as error cause*: 18.0 (3.2)<br>• *Disclosure responsibility*: 14.4 (2.9)<br>• *Team functioning*: 12.9 (1.2)<br>• *Patient involvement in reducing error*: 7.9 (3.3)<br>• *Importance of patient safety in the curriculum*: 16.6 (2.1)<br>• Attitudes to patient safety mean total score: 135.16 (10.6)			• Significantly higher scores among women than men in the subscores *Disclosure responsibility* (p<0.001) and *Team functioning* (p=0.029)
**Gropelli & Shanti, 2018 [36]**	United States of America	Junior and senior baccalaureate nursing students	N=196	Perception of safety and communication (Patient Safety Survey)	'Agree' attitude:<br>• 'Mistakes are held against respondents/nurses': 39% and 46%, respectively<br>• 'Believe nurses and students discuss safety issues': 79%<br>• 'Have a safety focus for their patients': 97%<br>• 'Policies and procedures are good at preventing errors': 93%<br>• 'Believe they are providing a safe care': 98%<br>• 'Are afraid to speak up if they see something wrong that could negatively affect the patient': 36%<br>• 'Students and nurses never, rarely, or sometimes discuss ways to prevent errors from happening in the unit': 44%<br>• 'Afraid to ask questions if something does not seem right': 20%<br>• 'Students are never, rarely, or sometimes informed about errors that happen during the semester': 53%<br>• 'Would never, rarely, or sometimes report an error that could harm the patient but does not': 33%<br>• 'Are concerned by errors': 95%<br>• 'Think that nurses are concerned about errors': 93%<br>• 'Felt that the clinical instructor was supportive of the student when an error occurred': 82%	• Significantly higher mean scores of the items 'As a student, I have a safety focus for my patient' (p<0.020), 'My patient has a safety focus for my shift' (p<0.028), 'My clinical instructor focuses on safety issues' (p<0.039), and 'Students are informed about errors that happened during the semester' (p<0.021) in junior level students than senior level students		
**Kamran et al., 2018 [24]**	Pakistan	Final-year medical students	N=122	Awareness of patient safety and attitudes (APSQ-III)	Scores (mean, SD):<br>• *Patient safety training received*: 5.72 (2.5)<br>• *Error reporting confidence*: 5.09 (3.7)<br>• *Working hours as error cause*: 6.04 (3.0)<br>• *Error inevitability*: 4.99 (2.9)<br>• *Professional incompetence as error cause*: 2.59 (3.5)<br>• *Disclosure responsibility*: 3.00 (3.3)<br>• *Team functioning*: 6.17 (1.4)<br>• *Patient involvement in reducing error*: 5.62 (1.9)<br>• *Importance of patient safety in the curriculum*: 5.10 (2.2)			• No significant difference in awareness with respect to gender
**Kamran et al., 2019 [25]**	Pakistan	Fourth- and final-year medical students (medical school of the military set up)	N=166	Awareness towards patient safety (APSQ-III)	• Eight items regarding shorter shifts, regular breaks, number of working hours for doctors, teaching and learning of students about patient safety, teamwork skills and competent doctors make errors rated by the majority of students with the best score (6.0)<br>• Second-highest item mean score (5.5): 'All errors should be reported'<br>• Best domain mean scores: *Working hours as an error cause* and *Team functioning* (6.0)<br>• Lowest domain mean scores: *Disclosure responsibility* (4.4), *Professional incompetence as an error cause* (4.5), *Error inevitability* (4.7)	• No significant differences with respect to academic year		• No significant differences with respect to gender

					• 'Human error is inevitable': 78% of participants agreed<br>• 'A true professional does not make mistakes': 41% disagreed (lowest score of 2.9)<br>• Second-lowest score: 'It is not necessary to report errors which do not result in adverse outcomes for the patient' (3.4)			
**Kiesewetter et al., 2014 [37]**	Germany	Medical students of all course years of the program	N=167	Attitudes towards patient safety (APSQ-adapted)	• Significantly lower values in the domain *Error reporting confidence* reported by students who already had attended courses on medical error and patient safety than by those without training<br>• Significantly lower values on *Error reporting confidence* reported by those who have made a medical error in the past (p<0.000)<br>• No significant influence of a pre-existing training in another medical field prior to the start of medical education on the knowledge and attitude on patient safety and medical errors	• Lower values on the scale *Error reporting confidence* reported by final year students than by first, second, third, fourth, and firth year students (p<0.000)		
**Kong et al., 2019 [38]**	China	Postgraduate nursing students	N=231	Attitude towards patient safety (C-SAQ)	• Highest score for *Stress recognition* (70.75±19.32), lowest score for *Working conditions* (65.22±17.83).<br>• 51.5% held positive attitudes towards *Stress recognition*, 45.5% towards *Perception of management*, 40.3% towards *Job satisfaction*, 39% towards *Teamwork climate*, 35.9% towards *Working conditions* and 30.7% towards *Safety climate*<br>• Students with work experience: Significantly higher scores in *Teamwork climate* (p<0.001), *Perception of management* (p<0.001) and *Job satisfaction* (p=0.002)<br>• Students with lower workload: significantly higher scores in *Safety climate* (p=0.035), *Job satisfaction* (p=0.006) and *Working conditions* (p=0.012); significantly lower scores for *Stress recognition* (p=0.026)<br>• Students who received patient safety education: significantly higher scores in all domains except for *Stress recognition*<br>• Positive correlations between domains scores (except for *Stress recognition*) and 'good collaboration with nurses and physicians', 'adhering to aseptic operating procedures', 'reporting adverse events' and 'actively learning about patient safety' (p<0.05)	• No significant differences for grade (i.e., 1st, 2nd, 3rd grade)		• No significant differences for gender
**Lee et al., 2018 [39]**	South Korea	Third-year undergraduate medical students	N=194	Perceptions and intentions regarding patient safety (modified Medical Student Safety Attitudes and Professionalism Survey - MSSAPS)	'Agree' attitude:<br>• 'Follow standard operating procedures, guidelines, and protocols for the floor and operating room' (*Safety Culture*): 73.7%<br>• 'The quality of care received by patients was impacted by teamwork during clinical rotation' (*Teamwork culture*): 78%<br>• 'It is easy to ask questions when there was something that they did not understand' (*Teamwork culture*): 40%<br>• 'Encouraged by colleagues to disclose errors to patient and families' (*Error Disclosure*): 10.3%<br>• 'Intention to encourage colleagues to tell patients and their families about medical errors' (*Error Disclosure*): 50.5%<br>• 'Think their hospital is overall safe': 60%<br>• Items with lowest percentage of positive responses: 'I received appropriate feedback about my performance' (33%) and 'The clinical culture made it easy to learn from the errors of others' (34.5%)<br>• Highest rated domain: *Teamwork culture*<br>• Lowest rated domain: *Error disclosure culture*			
**Leong et al., 2008 [40]**	United States of America	Dental students	N=107	Patient safety culture and comparison with benchmark (HSOPSC - The	• Dental students scored above average among composite level benchmarks (responses from 20 Hospitals) in *Overall perceptions of safety* (63% positive responses), *Management support for patient safety* (66%), and *Teamwork across units* (60%)<br>• Students scored below average for benchmarks in *Frequency of adverse events reported* (37%), *Organizational learning/Continuous*			

				Agency for Healthcare Research and Quality Hospital Survey on Patient Safety Culture)	*improvement* (55%), *Communication openness* (42%), *Feedback and communication about errors* (38%), *Non-punitive response to errors* (35%)<br>• Results in line with average for benchmark in *Supervisor expectations and actions promoting patient safety* (67%), *Teamwork within units* (74%), *Staffing* (54%), and *Handoffs and transitions of patients* (46%)			
**Leung & Patil, 2010 [41]**	Hong Kong	Fourth-year medical students	N=96	Perceptions (and knowledge) on patient safety issues (adapted questionnaire)	'Agree' attitude:<br>• 'Making errors in medicine is inevitable': 72%<br>• 'What is considered as 'best care' may not always be provided': 64%<br>• 'Competent physicians do not make errors': 26%<br>• 'There is no need to address an error which has not harmed patients': 6%<br>• 'The most effective strategy to prevent errors is 'to work harder and be more careful': 88%<br>• 'Patient safety is an important topic': 90%<br>• 'I would like to receive further teaching on patient safety': 79%<br>• 'It is important to learn how to analyze the cause of an error': 90%<br>• 'It is important to learn how to disclose an error to a patient': 87%<br>• 'It is important to learn how to disclose an error to a faculty member': 68%<br><br>'Disagree' attitude:<br>• 'Most errors are due to non-physician–related factors': 60%<br>• 'Approve the practice of "non-disclosure': 63%<br>• 'A reporting system does not help to diminish future errors': 71%<br>• 'Only physicians can determine the causes of a medical error': 59%<br><br>Neutral stance:<br>• 'Physicians should tolerate uncertainty in patient care': 45%<br>• 'The culture of medicine is conducive to the constructive management of error': 52%			
**Liu et al., 2018 [42]**	China	First-through-fifth year medical students	N=2498	Perceptions of patient safety culture (APSQ-III)	• Overall highest positive response rates for domains *Patient safety training received* (84.9%), *Patient involvement in reducing errors* (84.7%)<br>• Overall highest positive response rates for items 'I have a good understanding of patient safety issues as a result of my undergraduate medical training' (85.2%) and 'Patients have an important role in preventing medical errors' (85.1%)<br>• Overall lowest positive response rates for domains *Professional incompetence as an error cause* (58.4%) and *Disclosure responsibility* (69.4%)<br>• Overall lowest positive response rates for items 'Medical errors are a sign of incompetence' (32.5%) and 'It is not necessary to report errors, which do not result in adverse outcomes for the patient' (60.8%)	• Significantly more positive perceptions of *Working hours as an error cause* among first- and second-year students<br>• Significantly more positive views of *Team functioning* among third year students<br>• High positive response rate for *Error inevitability* among fourth- and fifth-year students<br>• Significant differences ($p<0.05$) among cohort in their positive response rates for *Patient safety training received, Error reporting confidence, Working hours as an error cause, Error inevitability, Team functioning, Patient involvement in reducing errors,* and		

						Importance of patient safety in the curriculum		
**Nabilou et al., 2015 [43]**	Iran	Students of medicine, nursing, and midwifery	N=121	Perceptions of patient safety (and knowledge) (adapted questionnaire)	'Agree' attitude:<br>• 'Medical errors are unavoidable': 60%<br>• 'The provided care is not always the best possible care': 80%<br>• 'Most errors are due to things that physicians cannot do anything about': 64%<br><br>'Disagree' attitude:<br>• 'Health care providers do not make harmful medical errors': 64%<br>• 'Report errors after occurrence': >50%<br>• 'Medical errors mean imminent harm to the patient': 67%<br>• Negative opinions about medical error reporting: 73%<br><br>• Significant relationship between students' perceptions of patient safety and their attitudes towards patient safety education (r=0.344, p=0.01)	• No significant relationship between students' perception of patient safety and year of study	• Significantly greater interest in patient safety education among nursing students (p=0.0017)<br>• Significantly greater interest in learning patient safety topics among nursing and midwifery students than medicine students<br>• Significantly higher scores on perception towards patient safety obtained by nursing and midwifery students than by medical students	• Significantly greater interest in patient safety education among men (p=0.001)<br>• No significant relationship between students' perception of patient safety and their gender
**Nadarajan et al., 2020 [20]**	Malaysia	Medical students from all the five year course of the program	N=324	Attitudes toward patient safety (APSQ-III)	• Highest positive response for item 'Teaching teamwork skills will reduce medical errors' (94.8%)<br>• Lowest positive response for item 'Better multi-disciplinary teamwork will reduce medical errors' (63.6%)<br>• Students' attitude positive in six domains: *Safety Training* (85.2%), *Error Reporting* (76.3%), *Working Hours* (89.5%), *Error Inevitability* (86.1%), *Team Functioning* (94.6%), *Patient Involvement* (80.1%)<br>• Students' attitude less positive in three domains: *Disclosure Responsibility* (68.5%), *Professional Incompetence* (70.0%), *Safety Curriculum* (71.1%)	• Significant differences in *Error reporting* (p=0.001), *Professional incompetence* (p<0.001) and *Disclosure responsibility* (p=0.002) domains between year of study<br>• Significantly higher average percentage of positive responses for *Error reporting* among third-year students than second-year students<br>• Significantly higher average percentage of positive responses for *Professional incompetence* among fifth-year than first-year students<br>• Significantly higher average percentage of positive responses for *Disclosure responsibility* among first-year than second-year students		• Significant differences between gender in perceptions on *Error reporting confidence* (p=0.002) (higher in men) and *Professional incompetence* (p=0.012) (higher in women)
**Park et al., 2019 [44]**	South Korea	Third- and fourth-year medical students	N=300	Attitudes about patient safety (APSQ-IV)	'Agree' attitude:<br>• 'Most harm to patients is unavoidable': 36.3%<br>• 'Satisfied with the patient-safety training they had received': 61.7%<br>• 'It is important for undergraduate medical students to receive formal			

					instruction on patient safety': 77.3%<br>• 'Patient safety could be learned through clinical experience after qualification': 41.3%<br>• 'It is effective to learn about patient safety issues before becoming qualified health professionals': 31.3%<br>• 'Confident about error reporting': 69%<br>• 'Junior team members should think carefully before initiating discussions about patient safety': 53.5%<br>• 'Human error is inevitable': 91.3%<br>• 'Medical errors are indicative of incompetence': 11.7<br>• 'Most medical errors resulted from careless health professionals': 33.7%<br>• 'Medical errors could be avoided by paying more attention while at work': 63%<br>• 'Remaining vigilant about potential risks could actually be detrimental to patient safety': 72%			
**Parry et al., 2009 [45]**	United States of America	Pediatric residents and fellows	N=209	Attitudes towards patient safety (SAQ and ad hoc survey)	SAQ<br>• Responses most positive in domains *Job satisfaction* (mean factor score=77.5), *Safety climate* (76.1), *Working conditions* (75.6), *Perception of management* (70.4)<br>• Responses less positively in domains *Teamwork climate* (64.6) and *Stress recognition* (59.1)<br><br>Ad hoc survey:<br>• Responses most positive in the areas of *clinical supervision and support* (75.0), *communication with their immediate senior physician* (65.5) and *orientation of new personnel* (63.9)<br>• Responses less positive in the areas of *handoffs and multiple services*, (58.1), *role identification during codes* (51.0), and *support following an adverse event* (42.8)			
**Shah et al., 2015 [46]**	Pakistan	Undergraduate medical students, house officers, and residents (Obstetrics and Gynecology, Surgery, and Medicine)	N=226	Perceptions about patient safety issues (and knowledge) (adapted questionnaire)	'Agree' attitude:<br>• 'Medical errors are inevitable': 57%<br>• 'Best care is not always provided to patients': 80.5%<br>• 'Competent physicians do not make errors': 46.9%<br>• 'Most errors are not related to physicians': 41.6%<br>• 'Medical errors should be reported': 73.9%<br>• 'There is no need to report a near miss event': 23.5%<br>• 'Only physicians can determine the cause of medical errors': 39.8%<br>• 'Reporting systems do little to reduce future errors': 90.3%<br>• 'Errors can be prevented by working hard and being more careful': 30.5%<br>• 'Uncertainty should not be tolerated in patient care': 78.3%<br>• 'The culture of medicine is supportive for dealing with errors': 44.7% (high uncertainty regarding this item)<br>• 'Patient safety is an important topic': 96%<br>• 'Patient safety should be taught in medical school:' 89.4%<br>• 'Would like to learn how to help friends respond to error': 85.8%<br>• 'Would like to learn how to analyze errors': 91.2%<br>• 'Would like to learn how to disclose errors to patients': 87.2%<br>• 'Would like to learn how to disclose errors to faculty': 89.8%	• Significantly higher proportion of students and house officer than residents wrongly agreeing with 'There is no need to report a near miss event' ($p=0.01$)<br>• Significantly higher proportion of undergraduates wrongly agreeing to 'Only physicians can determine the cause of medical errors' ($p<0.001$)<br>• Significantly higher proportion of residents agreeing to 'Most errors are not related to physicians' ($p=0.04$)		
**Sorokin et al., 2005 [47]**	United States of America	Residents and fourth-year medical students	N=321	Beliefs about adverse events, preventive strategies and view of disclosure (ad hoc	'Agree' attitude:<br>• 'Adverse events are preventable': 61%<br>• 'Feel the training prepares to prevent adverse events': 74%<br>• 'Many adverse events result from physician error': 31%<br>• 'Many adverse events result from nursing error': 38%<br>• 'Better teamwork with nurses would reduce adverse events': 88%	• 54% of participants in their first and second postgraduate year that Computerized Patient Order Entry was beneficial but only 36% of the upper-level		

				questionnaire)	• 'Advocate formal teaching of teamwork': 58%<br>• 'Physician vigilance is the best way to protect patients': 44%<br>• 'Improved sign-out and better procedural training to circumvent adverse events': 70%<br>• 'A lighter nursing load would deter adverse events': 66%<br>• 'Malpractice fears hinder the discussion of adverse patient events': 60%<br>• 'Responsibility to disclose adverse events to patients': 83%<br>• 'Feel obligated to notify risk management of such events': 60%	residents agreed (p=0.01)<br>• 37% of participants in their third and fourth postgraduate year agreed the 80-hour workweek would reduce adverse events, compared with the 59% agreement of the first and second postgraduate year group and 48% of the medical students (p=0.03)<br>• 71% of the third and fourth postgraduate year group reported comfort in disclosure discussions compared with 44% of medical students and 49% of the first and second postgraduate year group (p<0.01)		
**Tegegn et al., 2017 [48]**	Ethiopia	Fourth- and fifth- year pharmacy students	N=83	Attitudes towards patient safety (Patient Safety/Medical Fallibility Curriculum survey)	'Agree' attitude:<br>• 'After an error occurs, an effective strategy is to work hard to be more careful': 80.7%<br>• 'Peer-led education can help the understanding of patient safety concepts': 80.7<br>• 'If they see an error that did cause harm, they would not keep it to themselves': 81.9%<br>• 'Pharmacists should routinely spend part of their professional time working to improve patient care': 79.6%<br><br>'Disagree' attitude:<br>• 'Pharmacists should discuss and report errors to an affected patient and their family even if the patient is not harmed': 48.2%	• No significant association with year of study		• No significant association with gender
**Teigland et al., 2013 [49]**	United States of America	Medical students	N=450	Attitudes about patient safety education (ad hoc questionnaire)	• Mean score of 'Importance of patient safety knowledge compared to basic knowledge': 3.7 out of 5 (SD 0.90)<br>• Mean score of 'Importance of patient safety knowledge compared to clinical knowledge': 3.1 out of 5 (SD 0.79)<br>• Mean score of 'Importance of quality improvement knowledge compared to basic science knowledge': 3.2 out of 5 (SD 0.88)<br>• Mean score of 'Importance of quality improvement knowledge compared to clinical knowledge': 2.7 out of 5 (SD 0.76)<br>• Mean score of 'Real-life examples of mistakes presented by a physician or by patients, respectively, as most helpful method of learning about patient safety': 4.5 out of 5 (SD 0.7) and 4.1 out of 5 (SD 1.0)<br>• Patient safety education to be taught during clinical rotations preferred by 47%<br>• Patient safety education to be taught during the clinical skills class during first and second year preferred by 27%			
**Wetzel et al., 2012 [26]**	United States of America	Fourth-year medical students	N=139	Attitudes towards patient safety (APSQ-III)	• Attitudes supportive of patient safety reported by 75.7%<br>• Patient safety curricula considered as priority at the undergraduate level by 80.1%<br>• Positive responses to items about patient safety training by 83.5%			

					• Contribution of multidisciplinary teamwork to error reduction recognized by 88.8%<br>• Understanding of error inevitability reported by 92.8%<br>• Involving the patient in their care can reduce error: 87.4% agreed<br>• Desired behavior (i.e., reporting to a resident/intern or faculty member) after being involved in an error/near miss shown by 65.2%<br>• Observation of health care personnel deviate from safe medical practices reported by 85.6%<br>• 46% of respondents would always or often inform fellows of this deviation from safe practices, 24.5% would inform nurses, 17.8% residents/interns, 10.3% faculty members)			
**Yoshikawa et al., 2013 [50]**	Brazil	Third- and fourth-year nursing students and fourth- and fifth-year medical students	N=109	Comprehension and attitudes about patient safety (ad hoc questionnaire)	• 87.2% communicated the professors about the presence of conditions that can lead to the occurrence of errors in the training settings<br>• 74.3% notified the professor, manager or person in charge of the training site when an error occurred<br>• 75.2% notified the colleagues when an error occurred<br>• Mixed results regarding the attitude of communicating the error occurrence to the patient and family (agree 39.4%, disagree 33.9%) and if there is no damage (agreed 34.8%, disagreed 47.7%)<br><br>'Disagree' attitude:<br>• 'Systems to report errors make little difference in reducing future errors': 91.7%<br>• 'Only doctors can determine the cause of the occurrence of errors': 93.6%<br>• 'Students always perform internship activities in locations that promote good practices': 72.5%<br>• 'Whenever they identify situations that require improvements, they get the support of the institution for implementing safety measures': 62.4%			

Supplementary File 7. Characteristics of administered questionnaires.

Questionnaire	Version	Version validated in original language (yes/no)	Version validated in language in which it was administered (Yes/No/Information not available)		Total sample size*	Domains (items) (N)	Score system	Presentation of results
SAQ	Original version developed by the Agency for Healthcare Research and Quality	Yes	AlOlayan et al. [22]:<br>Parry et al. [45]	Yes<br>Yes	923	6 (36)	• 5-point Likert scale from 1 (strongly disagree) to 5 (strongly agree)<br>Positive answers: Likert 4/5, Neutral answers: Likert 3, Negative answers: Likert 1/2	• Mean Likert score (SD) by domain [22,30,38,45]<br>• N (%) of positive answer by domain [38]<br>• N (%) of negative, neutral, and positive answer by item [22,30]
	Customized	Yes	Al-Surimi et al. [30]	Not available		6 (36)		
	Chinese version (C-SAQ)	Yes	Kong et al. [38]	Yes		6 (31) (C-SAQ)		
APSQ	Version III	Yes	Alwhaibi et al. [13]<br>Escher et al. [35]<br>Kamran et al. [24]<br>Kamran et al. [25]<br>Liu et al. [42]<br>Nadarajan et al. [20]<br>Wetzel et al. [26]	Not available<br>Yes<br>Not available<br>Not available<br>Not available<br>Not available<br>Yes	4269	9 (26)	• 5-point Likert scale from 1 (strongly disagree) to 5 (strongly agree) [13,20,26,42]<br>Positive answers: Likert 4/5<br>Neutral answers: Likert 3<br>Negative answers: Likert 1/2<br>• 7-point Likert scale from 1 (strongly disagree) to 7 (strongly agree) [23–25,35, 37,44]<br>Positive answers: Likert 5/6/7	• Mean Likert score (SD) by domain [24,25,35,37]<br>• Mean Likert score (SD) by item [20,23–25, 37,44]<br>• N (%) of negative, neutral, and positive answer by item [13,20]<br>• N (%) of positive answer/agreement by domain and item [42,44]<br>• % responding with desired

	Version IV	Yes	Bari et al. [23];<br><br>Park et al. [44]	Not available<br><br>Not available		10 (29)	Neutral answers: Likert 4<br>Negative answers: Likert 1/2/3	attitude by item [26]<br><br>• Score of key factor domain [24]
	Adapted version	Yes	Kiesewetter et al. [37]	Yes		6 (14)		
HSOPSC	Original	Yes	Bump et al. [27]<br><br>Bump et al. [28]<br><br>Leong et al. [40]	Yes<br><br>Yes<br><br>Yes	1988	12 (42; 48 for Leong et al. [40]) (2 outcome measures)	• 5-point Likert scale from 1 (strongly disagree) to 5 (strongly agree)	• % of positive responses [40] or average % positive response [27,28] by domain
Other questionnaires	Thomas Jefferson Survey on Inpatient Adverse Events	No	De la Tassa et al. [33]	No	3591	20 items	• 5-point Likert scale from 1 (strongly disagree) to 5 (strongly agree) [29,31–34,36,39,41,43,46,47,50] (one being "not important/helpful" and 5 being "very important/helpful" in Teigland et al. [47]	• Mean Likert score (SD) by domain [39]<br><br>• Mean Likert score (SD) by item [39,49] (in Teigland et al. [49] also % of importance by item)<br><br>• N (%) of agreements/disagreements, of

	Junior Doctor - Patient Safety Attitudes and Climate Questionnaire	No	Durani et al. [34]	No		3 (40, of which 30 for attitudes to patient safety and safety climate)	• 4-point Likert scale [48]	'agree' attitude, of negative, neutral, and positive by item [29,31–34,39,41,43,46– 48,50]<br>• Mean score for clinical setting questions and % of agreement/disagreement by item (never/rarely/sometimes for communication and safety reporting domain) [36]
	Patient Safety Survey	No	Gropelli & Shanti [36] (1)	No		4 (33)		
	Medical Student Safety Attitudes and Professionalism Survey	Yes	Lee et al. [39]	Yes		5 (25)		
	Patient Safety/Medical Fallibility Curriculum Survey	Yes	Tegegn et al. [48]	Not available		21 items		
	Ad hoc or adapted** questionnaires	No	Almaramhy et al. [29]<br>Bowman et al. [31]<br>Cauduro et al. [32]<br>Leung & Patil, [41]<br>Nabilou et al. [43]<br>Shah et al. [46]<br>Sorokin et al. [47]<br>Teigland et al. [49]	No<br>No<br>No<br>No<br>No<br>No<br>No<br>No		/		

			Yoshikawa et al. [50]	No				

*considering only those subjects who participated in the survey and were considered for the purposes of this review
**other than adapted SAQ and APSQ

Supplementary File 8. Mean scores and percentages of positive answers per patient safety domain reported by studies applying SAQ, APSQ, and HSOPSC.

SAQ	Domains	Mean score 100 ±SD	Positive answers (%)
	Teamwork climate		
	AlOlayan et al., 2020 [22]	61.2	60.8*
	Al-Surimi et al., 2018 [30]	54.4 ± 28.0	48.9
	Kong et al., 2019 [38]	67.3 ± 16.8	39.0
	Parry et al., 2009 [45]	64.6 ± 12.5	/
	Safety climate		
	AlOlayan et al., 2020 [22]	59.7	56.0*
	Al-Surimi et al., 2018 [30]	51.4 ± 29.7	40.7
	Kong et al.2019 [38]	66.2 ± 15.5	30.7
	Parry et al., 2009 [45]	76.1 ± 16.3	/
	Job satisfaction		
	AlOlayan et al., 2020 [22]	70.3 ± 21.2	67.9*
	Al-Surimi et al., 2018 [30]	64.5 ± 33.8	65.6
	Kong et al., 2019 [38]	66.2 ± 19.7	40.3
	Parry et al., 2009 [45]	77.5 ± 19.4	/
	Stress recognition		
	AlOlayan et al., 2020 [22]	60.9	49.9*
	Al-Surimi et al., 2018 [30]	56.2 ± 37.8	49.8
	Kong et al., 2019 [38]	70.8 ± 19.3	51.5
	Parry et al., 2009 [45]	59.1 ± 17.1	/
	Perceived management support		
	AlOlayan et al., 2020 [22]	61.3	47.6*
	Al-Surimi et al., 2018 [30]	50.7 ± 37.7	44.8
	Kong et al., 2019 [38]	69.3 ± 16.6	45.5
	Parry et al., 2009 [45]	70.4 ± 20.0	/
	Working conditions		
	AlOlayan et al., 2020 [22]	64.7 ± 20.1	58.7*
	Al-Surimi et al., 2018 [30]	55.3 ± 32.1	57.0
	Kong et al., 2019 [38]	65.2 ± 17.8	35.9
	Parry et al., 2009 [45]	75.6 ± 15.6	/
**APSQ**	**Domains**	**Mean Likert Score ±SD (max Likert)**	**Positive answers (%)**
	Patient Safety Training received		
	Alwhaibi et al., 2020 [13]	/ (5)	59.6*
	Liu et al., 2018 [42]	/(5)	84.9
	Nadarajan et al., 2020 [20]	4.10 ± 0.77*(5)	85.2
	Wetzel et al., 2012 [26]	/ (5)	83.5
	Escher et al., 2017 [35]	5.2* (7)	/
	Kamran et al., 2018 [24]	5.72 ± 2.5 (7)	/
	Kamran et al., 2019 [25]	5.0 (7)	/
	Bari et al., 2017 [23]	5.64 ± 1.58* (7)	/
	Park et al., 2019 [44]	4.92 ± 1.45* (7)	63.7*
	Kiesewetter et al., 2014 [37]	3.76± 1.26 (7)	/
	Error reporting confidence		
	Alwhaibi et al., 2020 [13]	/ (5)	64.8*
	Liu et al., 2018 [42]	/ (5)	74.9
	Nadarajan et al., 2020 [20]	3.97 ± 0.87* (5)	76.3
	Wetzel et al., 2012 [26]	/ (5)	57.6
	Escher et al., 2017 [35]	4.9* (7)	/
	Kamran et al., 2018 [24]	5.09 ± 3.7 (7)	/
	Kamran et al., 2019 [25]	5.0 (7)	/
	Bari et al., 2017 [23]	4.92 ± 1.79* (7)	/
	Park et al., 2019 [44]	4.74 ± 1.38* (7)	59.3*
	Kiesewetter et al., 2014 [37]	4.08±1.38 (7)	/
	Working hours as error cause§		
	Alwhaibi et al., 2020 [13]	/ (5)	63.3*
	Liu et al., 2018 [42]	/ (5)	83.4
	Nadarajan et al., 2020 [20]	4.30 ± 0.77* (5)	89.5
	Wetzel et al., 2012 [26]	/ (5)	73.4
	Escher et al., 2017 [35]	6.0* (7)	/
	Kamran et al., 2018 [24]	6.04 ± 3.0 (7)	/
	Kamran et al., 2019 [25]	6.0 (7)	/
	Kiesewetter et al., 2014 [37]	6.11±1.11 (7)	/
	Error inevitability		
	Alwhaibi et al., 2020 [13]	/ (5)	59.1*
	Liu et al., 2018 [42]	/ (5)	74.9
	Nadarajan et al., 2020 [20]	4.00 ± 0.80* (5)	86.1

	Wetzel et al., 2012 [26]	/ (5)	92.8
	Escher et al., 2017 [35]	6.0* (7)	/
	Kamran et al., 2018 [24]	4.99 ± 2.9 (7)	/
	Kamran et al., 2019 [25]	4.7 (7)	/
	Bari et al., 2017 [23]	4.91 ± 1.78* (7)	/
	Park et al., 2019 [44]	6.16 ± 1.07* (7)	91.9*
	Kiesewetter et al., 2014 [37]	6.59±0.81 (7)	/
	Professional incompetence as error cause***		
	Alwhaibi et al., 2020 [13]	/ (5)	23.7*
	Liu et al., 2018 [42]	/ (5)	58.4
	Nadarajan et al., 2020 [20]	2.70 ± 0.77* (5)	70.0
	Wetzel et al., 2012 [26]	/ (5)	64.0
	Escher et al., 2017 [35]	6.0* (7)	/
	Kamran et al., 2018 [24]	2.59 ± 3.5 (7)	/
	Kamran et al., 2019 [25]	4.5 (7)	/
	Bari et al., 2017 [23]	5.24 ± 1.71* (7)	/
	Park et al., 2019 [44]	3.70 ± 1.44* (7)	63.9*
	Disclosure responsibility***		
	Alwhaibi et al., 2020 [13]	/ (5)	56.9*
	Liu et al., 2018 [42]	/ (5)	69.4
	Nadarajan et al., 2020 [20]	3.23 ± 0.97* (5)	68.5
	Wetzel et al., 2012 [26]	/ (5)	65.7
	Escher et al., 2017 [35]	5.2* (7)	/
	Kamran et al., 2018 [24]	3.00 ± 3.3 (7)	/
	Kamran et al., 2019 [25]	4.4 (7)	/
	Bari et al., 2017 [23]	4.75 ± 2.24* (7)	/
	Park et al., 2019 [44]	4.98 ± 1.40* (7)	77.0*
	Team functioning***		
	Alwhaibi et al., 2020 [13]	/ (5)	76.9*
	Liu et al., 2018 [42]	/ (5)	76.9
	Nadarajan et al., 2020 [20]	4.50 ± 0.65* (5)	94.6
	Wetzel et al., 2012 [26]	/ (5)	88.8
	Escher et al., 2017 [35]	6.6* (7)	/
	Kamran et al., 2018 [24]	6.17 ± 1.4 (7)	/
	Kamran et al., 2019 [25]	6.0 (7)	/
	Bari et al., 2017 [23]	5.88 ± 1.48* (7)	/
	Patient involvement in reducing error		
	Alwhaibi et al., 2020 [13]	/ (5)	66.0*
	Liu et al., 2018 [42]	/ (5)	84.7
	Nadarajan et al., 2020 [20]	4.20 ± 0.75* (5)	80.1
	Wetzel et al., 2012 [26]	/ (5)	87.4
	Escher et al., 2017 [35]	3.8* (7)	/
	Kamran et al., 2018 [24]	5.62 ± 1.9 (7)	/
	Kamran et al., 2019 [25]	5.3 (7)	/
	Bari et al., 2017 [23]	5.08 ± 1.84* (7)	/
	Park et al., 2019 [44]	5.70 ± 1.27* (7)	61.43*
	Kiesewetter et al., 2014 [37]	4.67±1.39 (7)	/
	Importance of patient safety in the curriculum		
	Alwhaibi et al., 2020 [13]	/ (5)	62.5*
	Liu et al., 2018 [42]	/ (5)	71.6
	Nadarajan et al., 2020 [20]	3.70 ± 0.80* (5)	71.1
	Wetzel et al., 2012 [26]	/ (5)	80.1
	Escher et al., 2017 [35]	5.7* (7)	/
	Kamran et al., 2018 [24]	5.10 ± 2.2 (7)	/
	Kamran et al., 2019 [25]	5.5 (7)	/
	Bari et al., 2017 [23]	5.27 ± 1.77* (7)	/
	Park et al., 2019 [44]	4.23 ± 1.62* (7)	55.8*
	Kiesewetter et al., 2014 [37]	4.97±1.39 (7)	/
	Patient safety general**		
	Bari et al., 2017 [23]	5.59 ± 1.53* (7)	/
	Park et al., 2019 [44]	4.81 ± 1.45* (7)	74.4*
	Situational awareness**		
	Bari et al., 2017 [23]	5.74 ± 1.50* (7)	/
	Park et al., 2019 [44]	5.66 ± 1.27* (7)	66.4*
**HSOPSC**	Overall perceptions of safety		
	Bump et al., 2015 [27]	/	55^
	Bump et al., 2017 [28]	/	55^
	Leong et al., 2008 [40]	/	63
	Frequency of adverse events reported		
	Bump et al., 2015 [27]	/	58^
	Bump et al., 2017 [28]	/	48^
	Leong et al., 2008 [40]	/	37
	Supervisor/manager expectations and actions promoting patient safety		

	Bump et al., 2015 [27]<br>*Bump et al., 2017 [28]*<br>*Leong et al., 2008 [40]*	/<br>/<br>/	80^<br>80^<br>67
	Organizational learning/Continuous improvements<br>*Bump et al., 2015 [27]*<br>*Bump et al., 2017 [27]*<br>*Leong et al., 2008 [40]*	 /<br>/<br>/	 61^<br>65^<br>55
	Teamwork within units<br>*Bump et al., 2015 [27]*<br>*Bump et al., 2017 [28]*<br>*Leong et al., 2008 [40]*	 /<br>/<br>/	 60^<br>60^<br>74
	Communication openness<br>*Bump et al., 2015 [27]*<br>*Bump et al., 2017 [28]*<br>*Leong et al., 2008 [40]*	 /<br>/<br>/	 51^<br>55^<br>42
	Feedback and communications about errors<br>*Bump et al., 2015 [27]*<br>*Bump et al., 2017 [28]*<br>*Leong et al., 2008 [40]*	 /<br>/<br>/	 47^<br>53^<br>38
	Nonpunitive response to errors<br>*Bump et al., 2015 [27]*<br>*Bump et al., 2017 [28]*<br>*Leong et al., 2008 [40]*	 /<br>/<br>/	 40^<br>40^<br>35
	Staffing<br>*Bump et al., 2015 [27]*<br>*Bump et al., 2017 [28]*<br>*Leong et al., 2008 [40]*	 /<br>/<br>/	 60^<br>60^<br>54
	Management support for patient safety<br>*Bump et al., 2015 [27]*<br>*Bump et al., 2017 [28]*<br>*Leong et al., 2008 [40]*	 /<br>/<br>/	 55^<br>62^<br>66
	Teamwork across units<br>*Bump et al., 2015 [27]*<br>*Bump et al., 2017 [28]*<br>*Leong et al., 2008 [40]*	 /<br>/<br>/	 56^<br>73^<br>60
	Handoff and transitions of patients<br>*Bump et al., 2015 [27]*<br>*Bump et al., 2017 [28]*<br>*Leong et al., 2008 [40]*	 /<br>/<br>/	 38^<br>45^<br>46

*calculated; **only in APSQ IV; §missing in APSQ IV; ***missing (not valid) in the adapted APSQ version and in APSQ version IV; ^estimated on the basis of the presented figures
